# Supplementary material for: Uremic toxin indoxyl sulfate induces trained immunity via the AhR-dependent arachidonic acid pathway in end-stage renal disease (ESRD)
Source: eLife. 2024 Jul 9;12:RP87316. doi: 10.7554/eLife.87316 (PMC11233136; doi:10.7554/eLife.87316)
Supplement: Figure 4—source data 2. [file elife-87316-fig4-data2.pdf]

Figure 4A, western blotting data

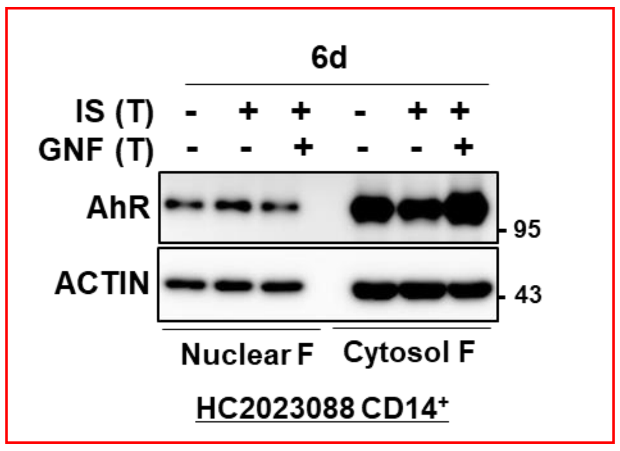

Figure 4A, left panel

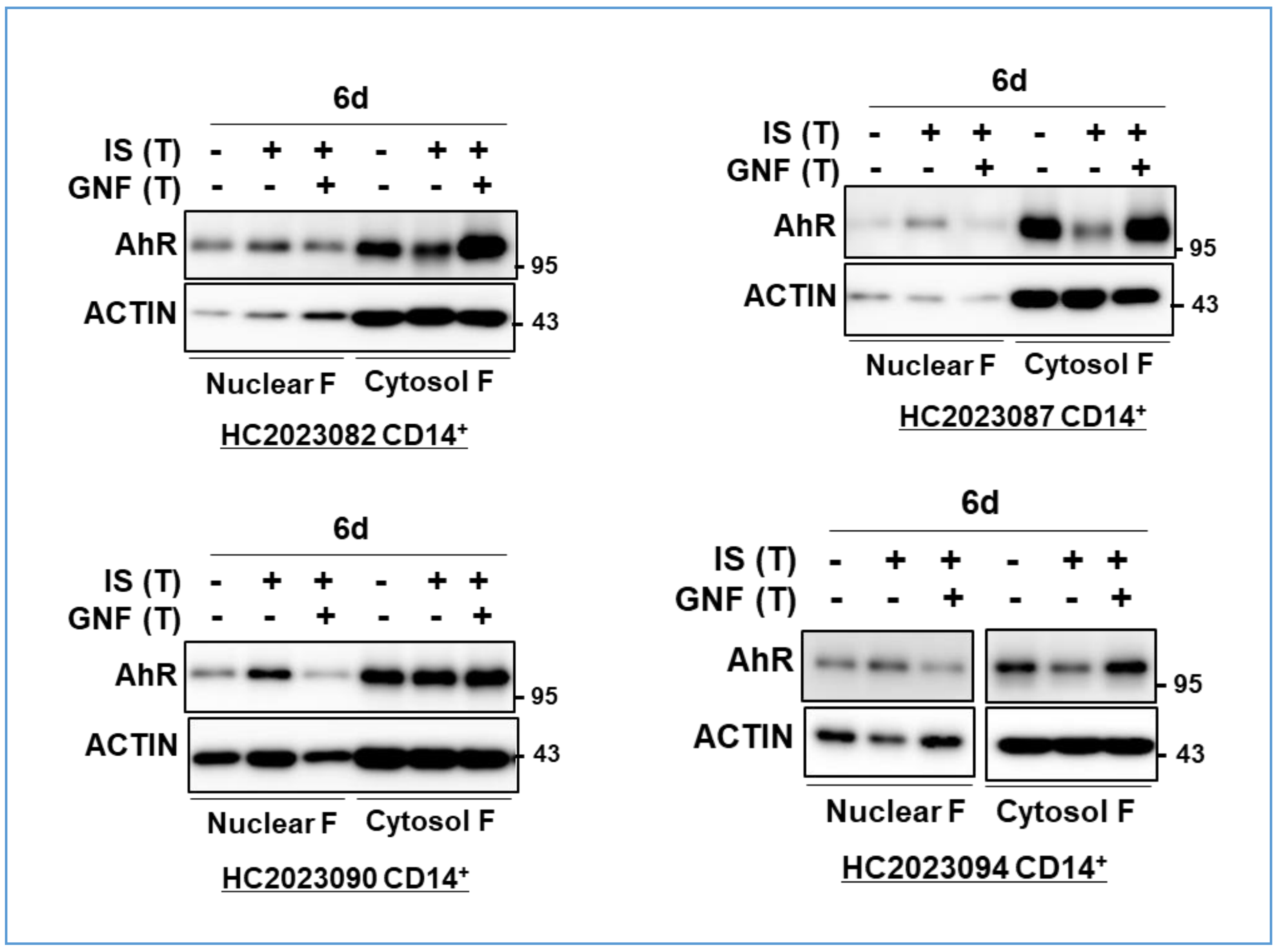

Figure 4A, right graph

Figure 4A, western blotting data

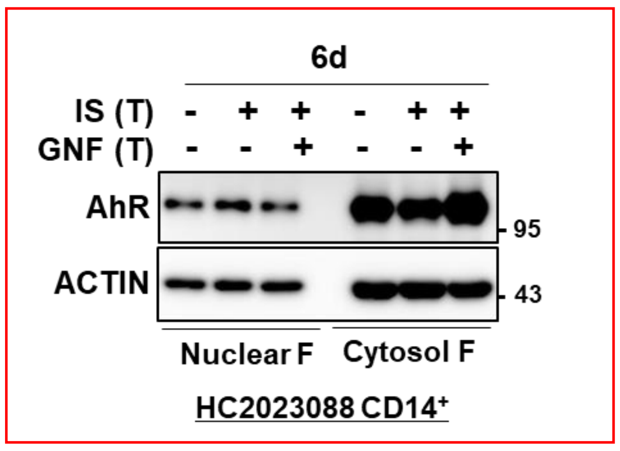

HC2023088 CD14+

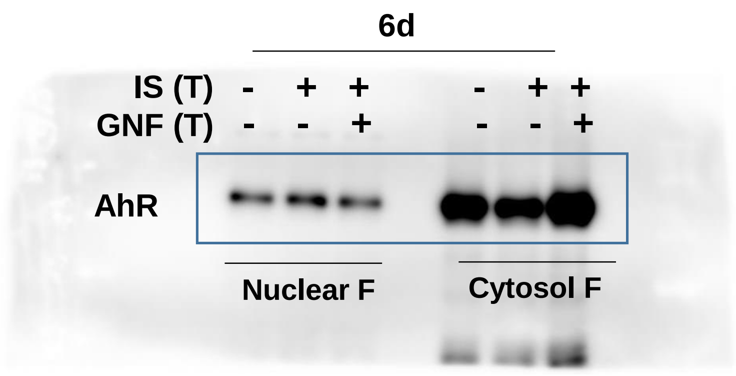

File name: AhR_HC2023088. jpg

AhR

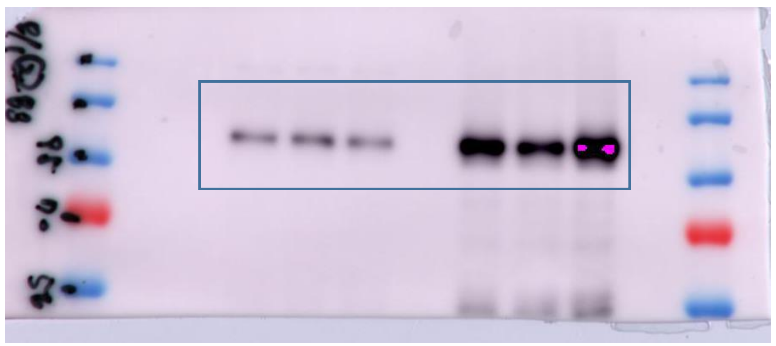

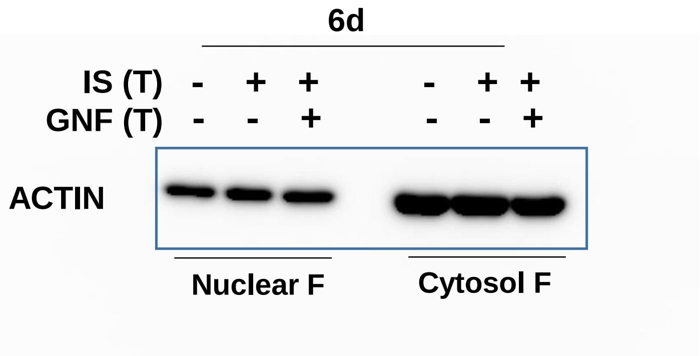

File name: Actin_HC2023088. jpg

ACTIN

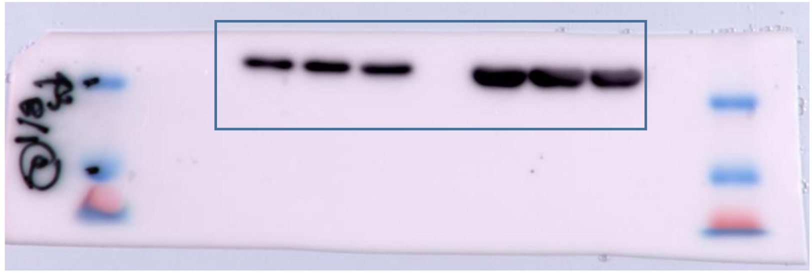

Figure 4A, western blotting data

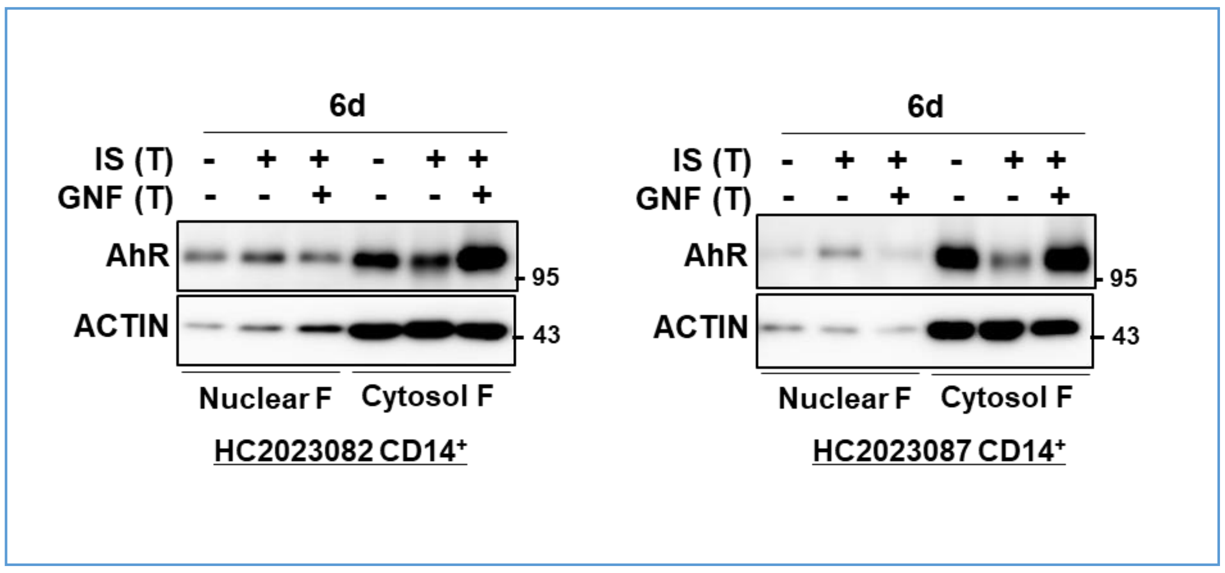

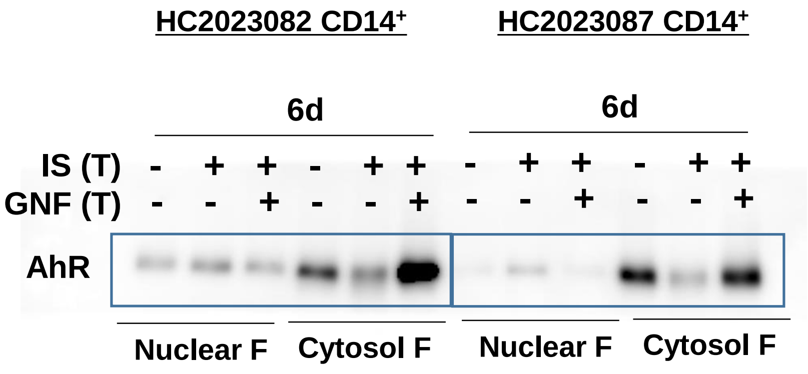

File name: AhR_HC2023082 87 90.jpg

AhR

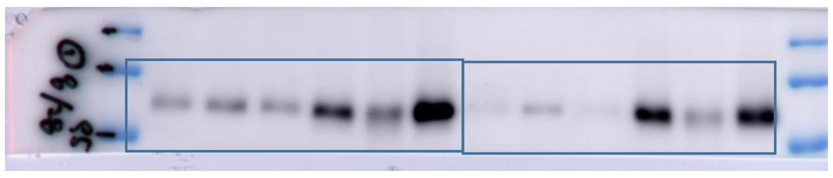

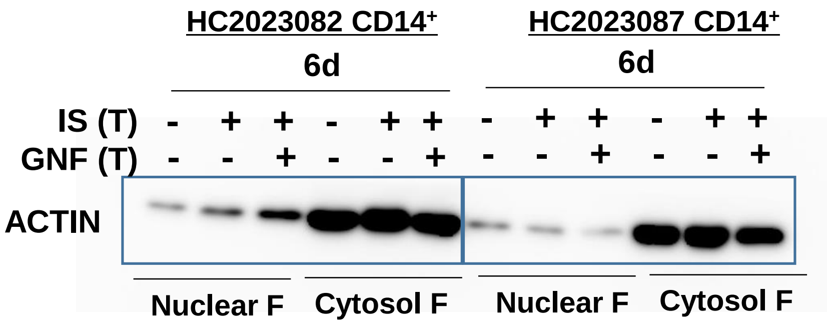

File name: Actin_HC2023082 87 90.jpg

ACTIN

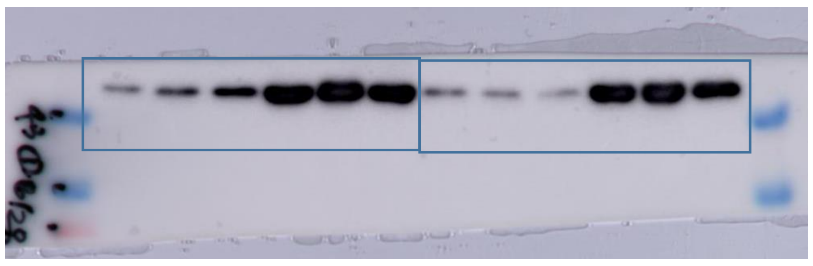

Figure 4A, western blotting data

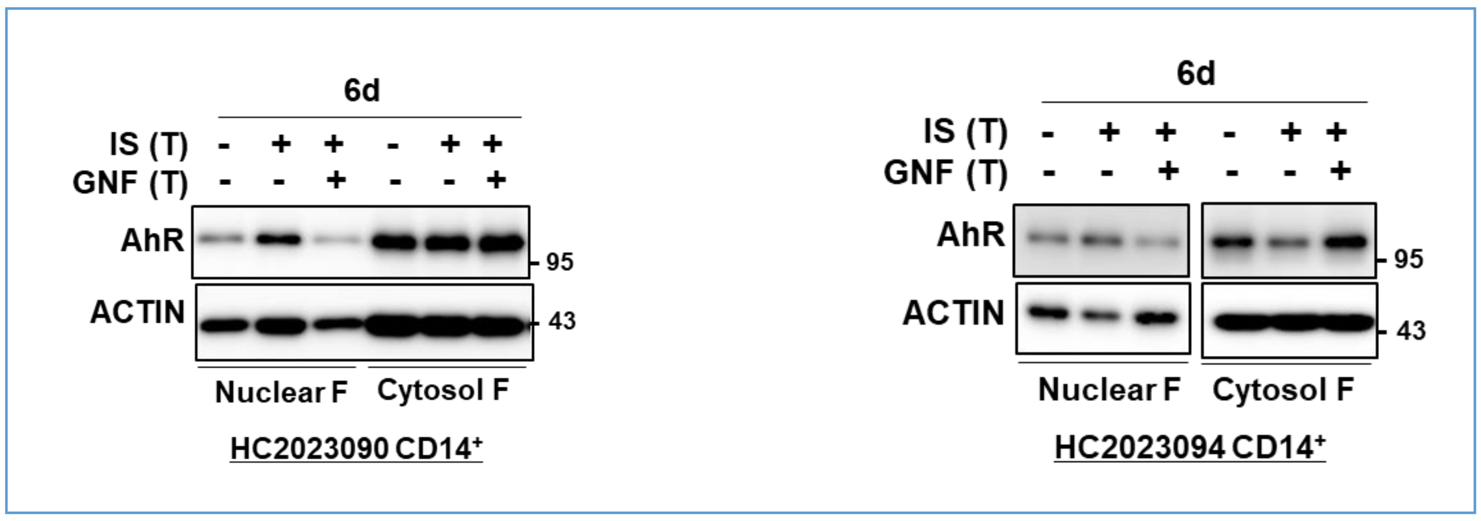

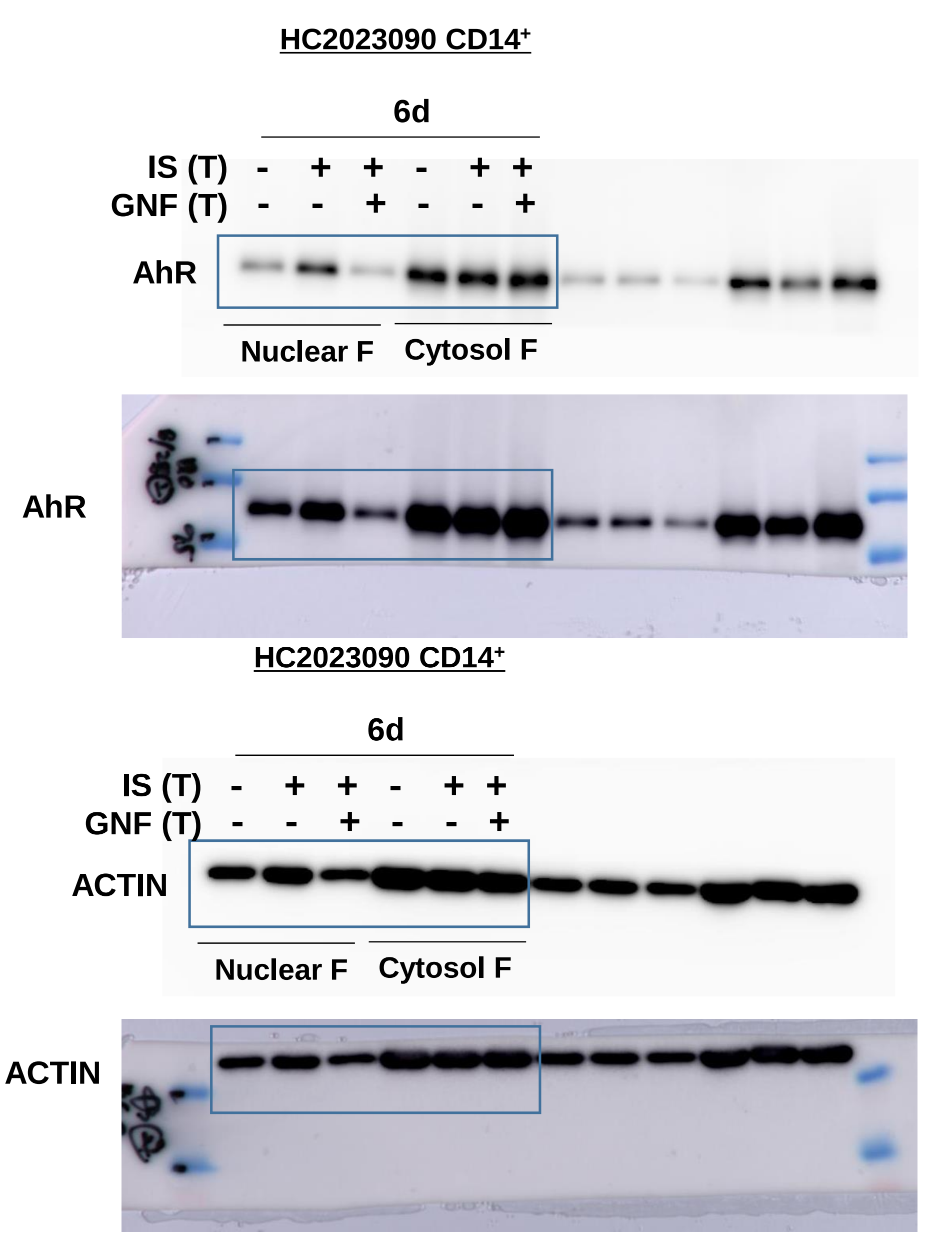

File name: AhR_HC2023082 87 90.jpg
Actin_HC2023082 87 90.jpg

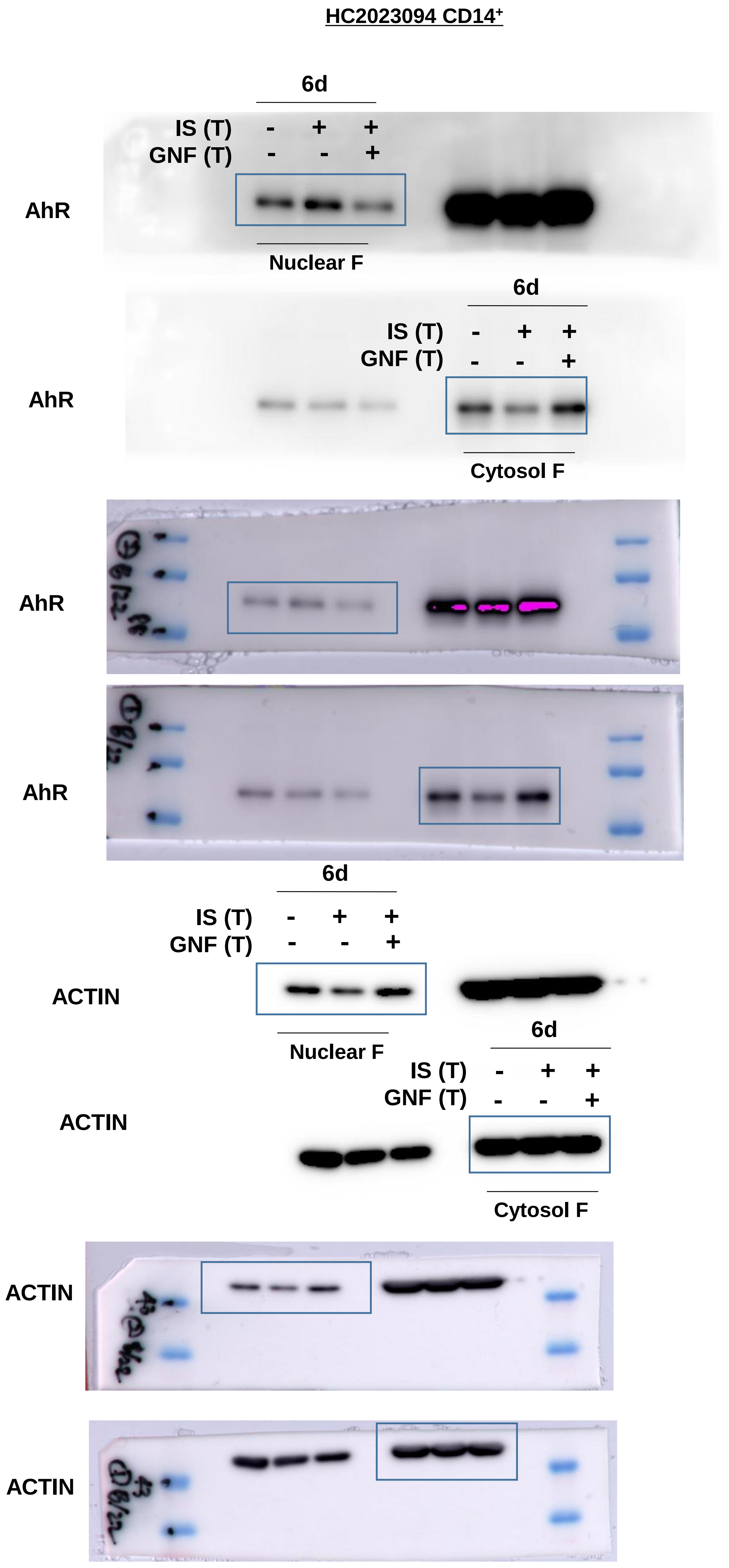

File name: AhR_HC2023094.jpg
Actin_HC2023094.jpg
