# Supplementary figures and images for: Uremic toxin indoxyl sulfate induces trained immunity via the AhR-dependent arachidonic acid pathway in end-stage renal disease (ESRD)
Source: eLife. 2024 Jul 9;12:RP87316. doi: 10.7554/eLife.87316 (PMC11233136; doi:10.7554/eLife.87316)

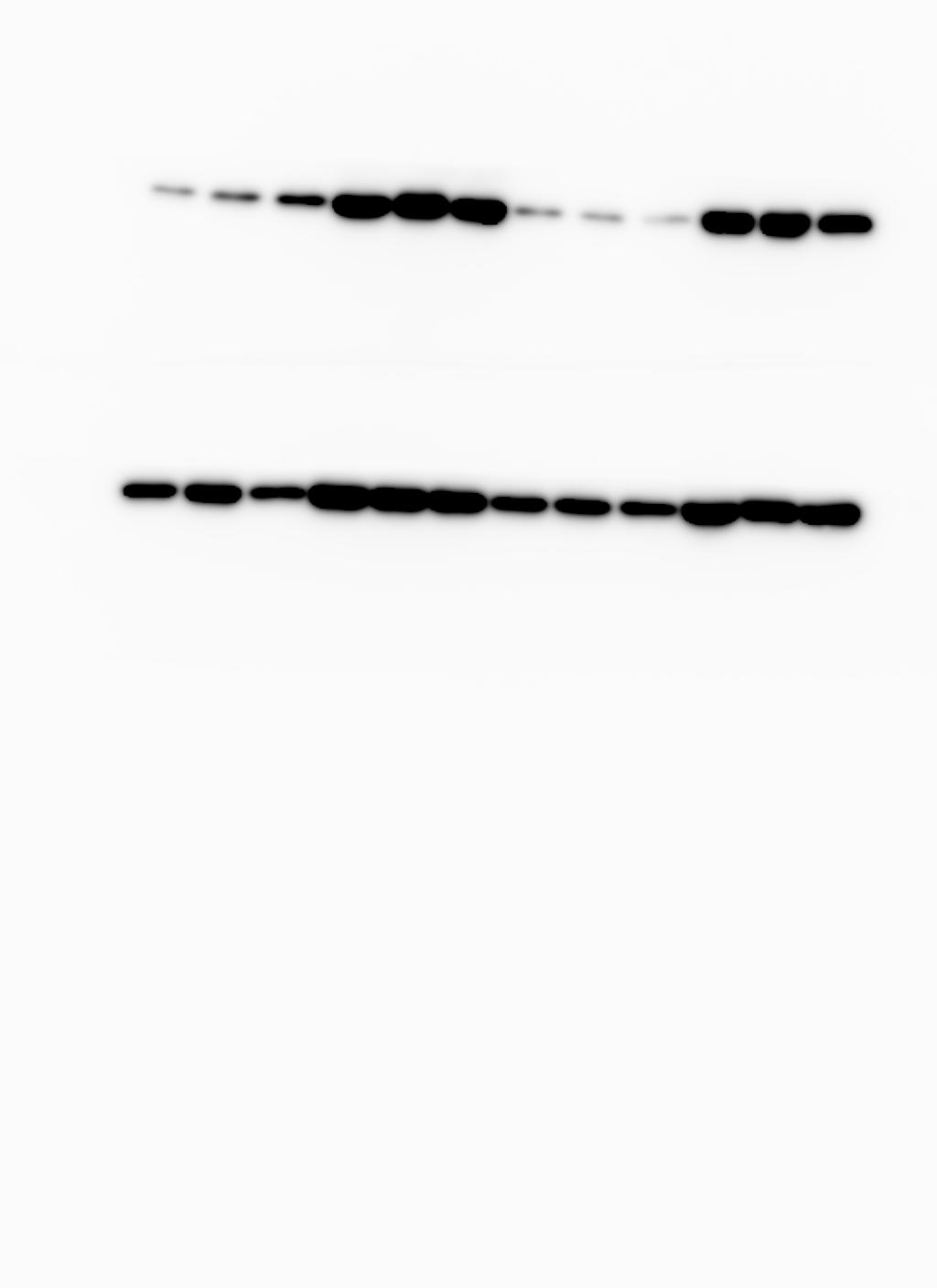

Supplement: Figure 4—source data 3. [file elife-87316-fig4-data3.zip › Actin_HC2023082 87 90.jpg]

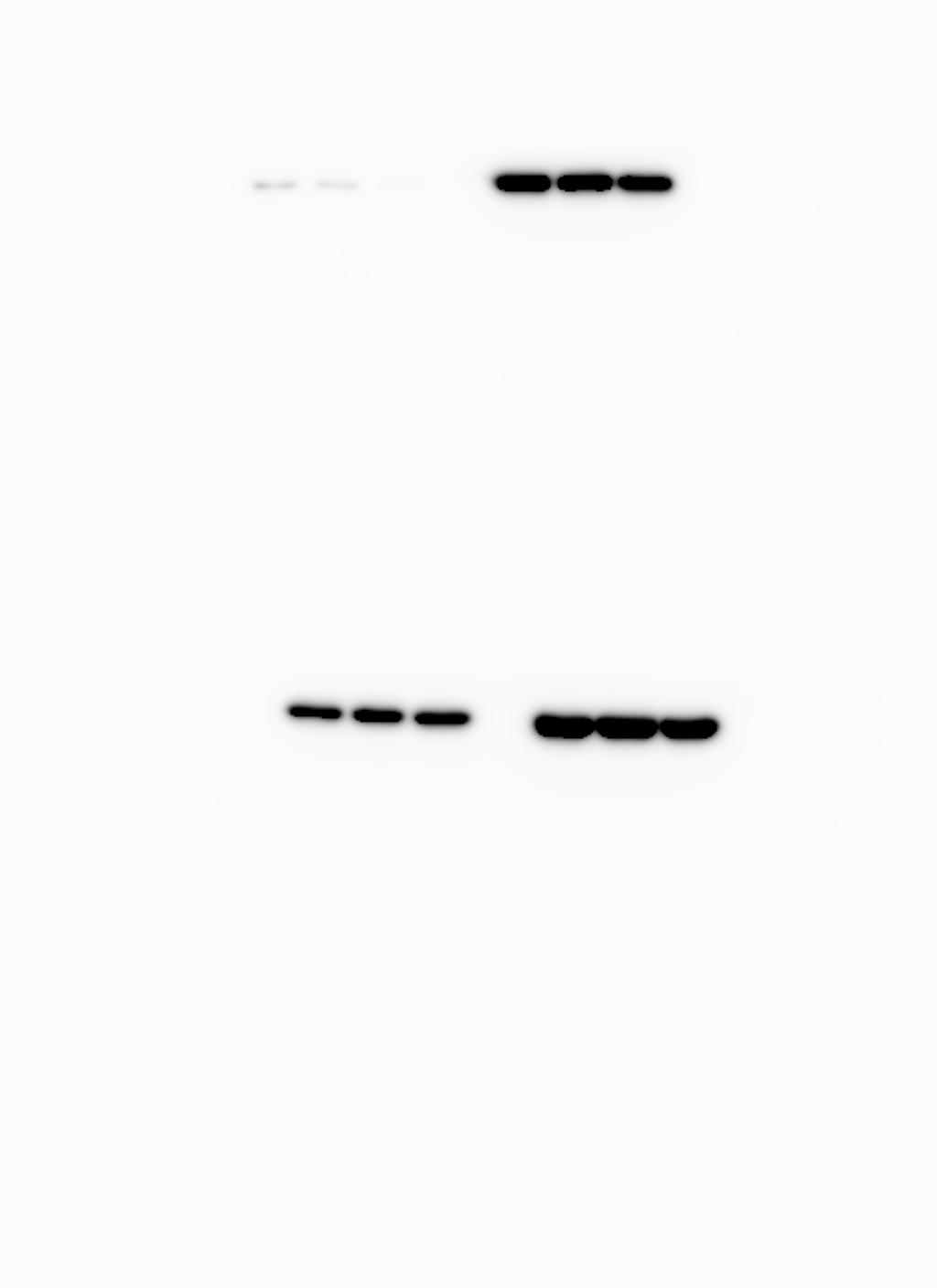

Supplement: Figure 4—source data 3. [file elife-87316-fig4-data3.zip › Actin_HC2023088.jpg]

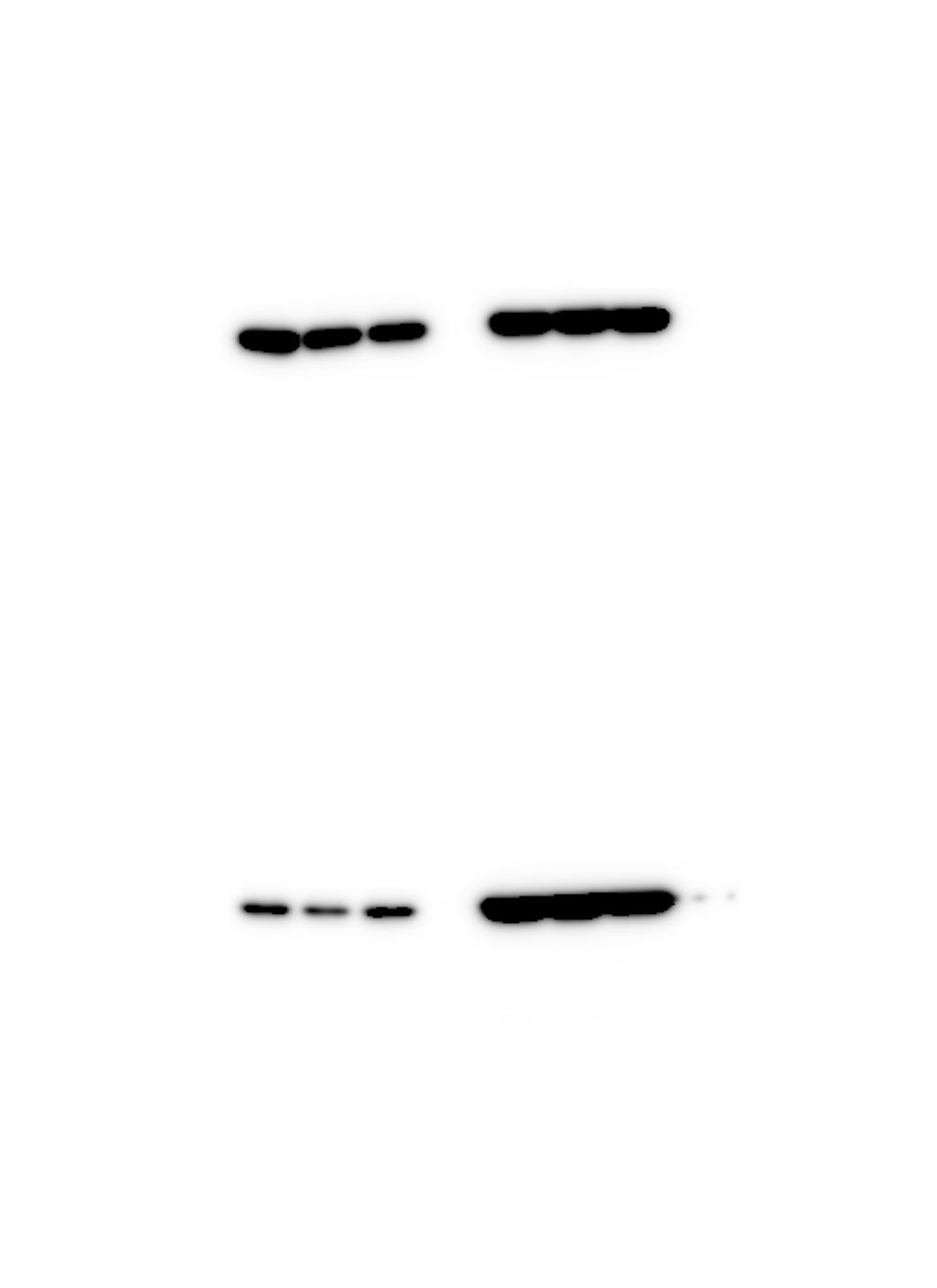

Supplement: Figure 4—source data 3. [file elife-87316-fig4-data3.zip › Actin_HC2023094.jpg]

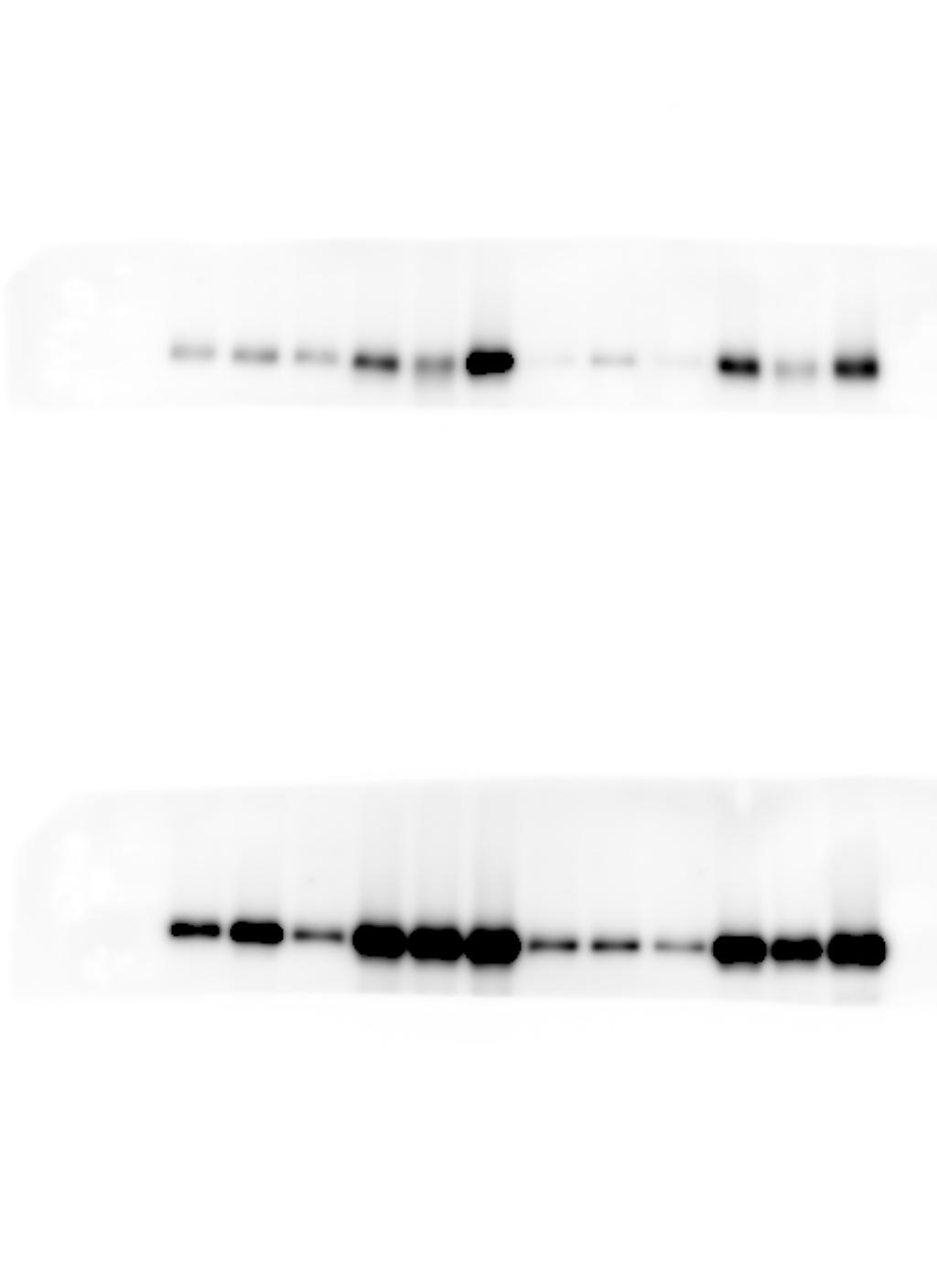

Supplement: Figure 4—source data 3. [file elife-87316-fig4-data3.zip › AhR_HC2023082 87 90.jpg]

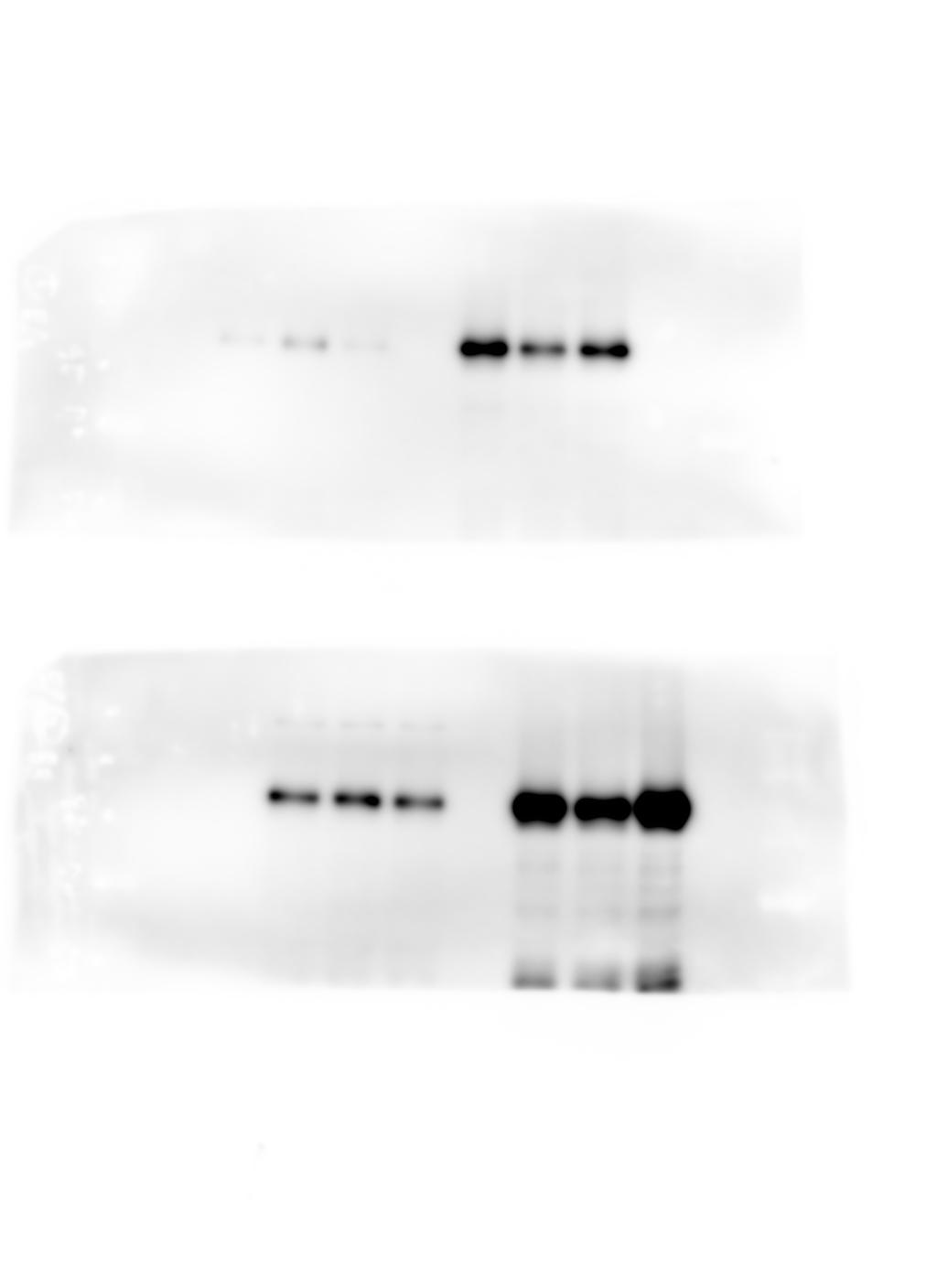

Supplement: Figure 4—source data 3. [file elife-87316-fig4-data3.zip › AhR_HC2023088.jpg]

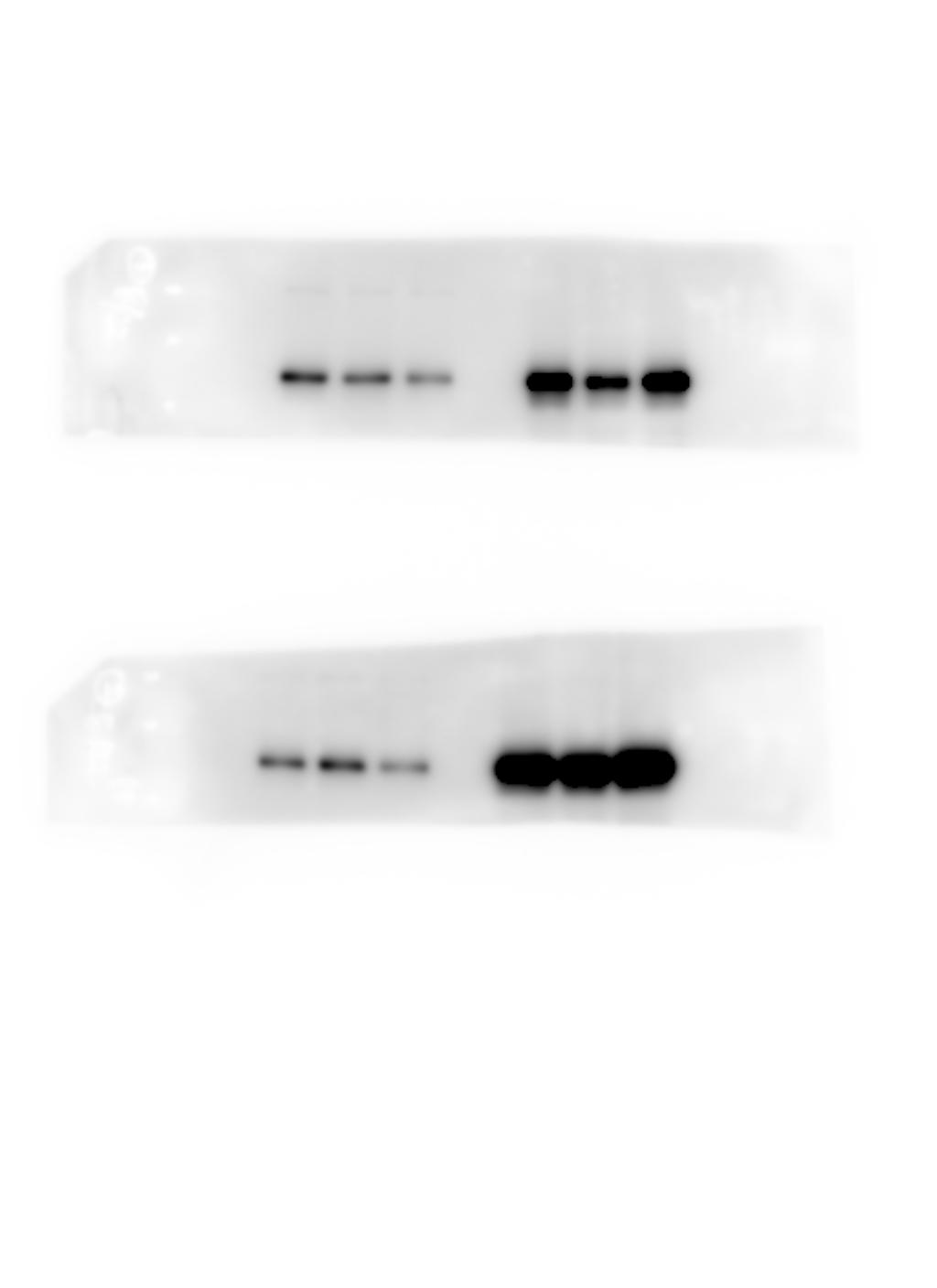

Supplement: Figure 4—source data 3. [file elife-87316-fig4-data3.zip › AhR_HC2023094.jpg]

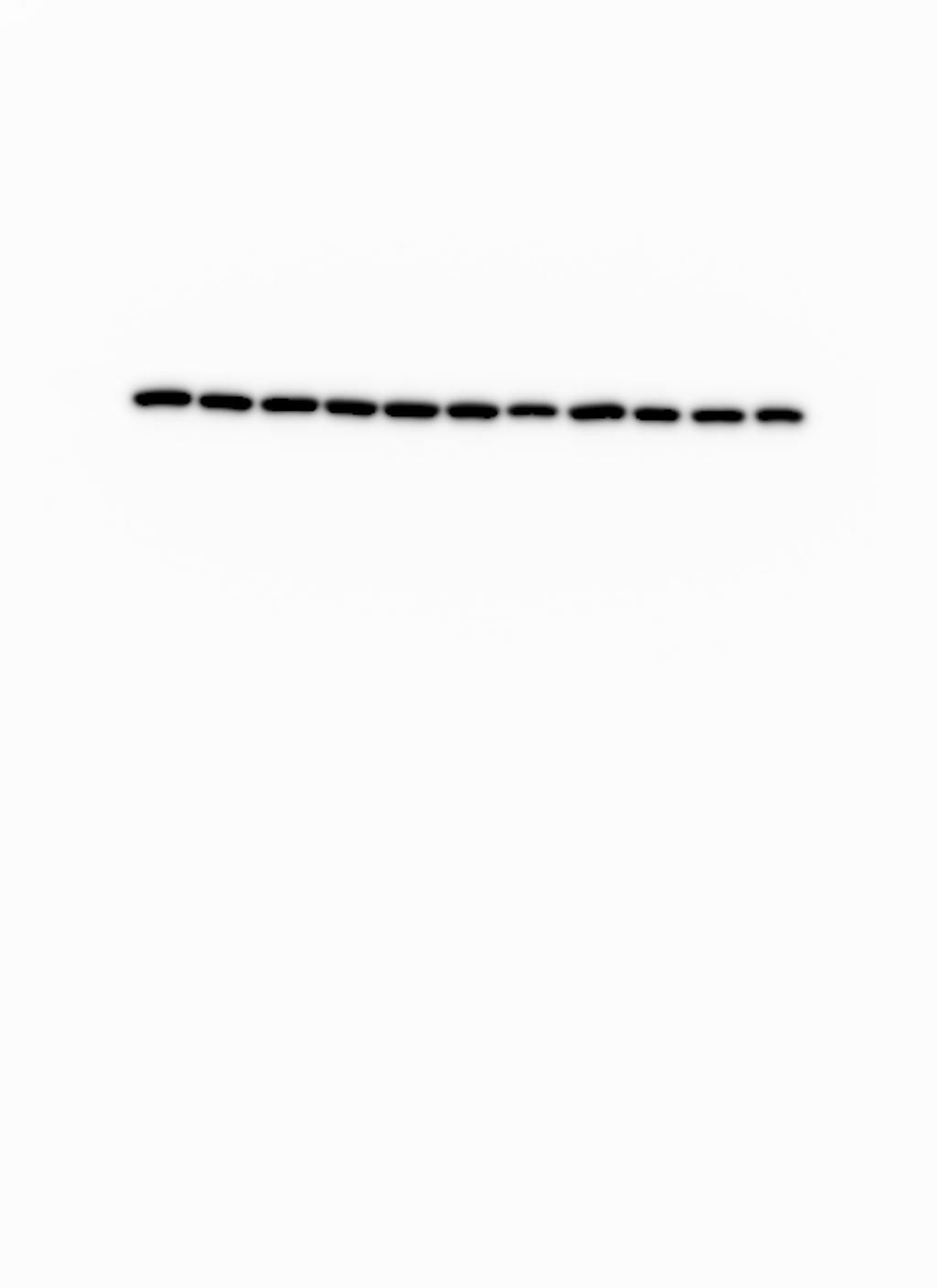

Supplement: Figure 4—figure supplement 1—source data 3. [file elife-87316-fig4-figsupp1-data3.zip › Actin_HC2022039.jpg]

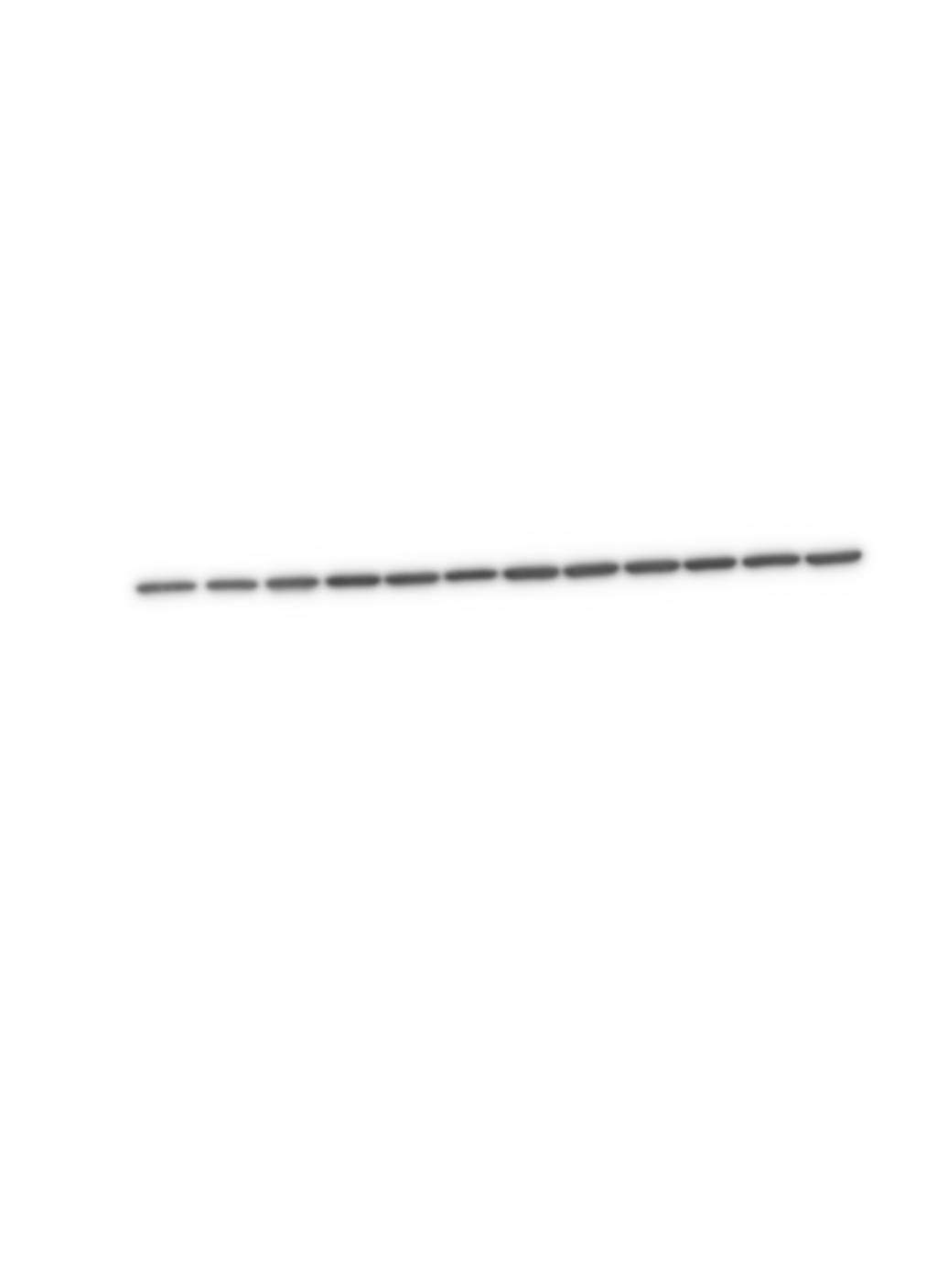

Supplement: Figure 4—figure supplement 1—source data 3. [file elife-87316-fig4-figsupp1-data3.zip › Actin_HC2022043.jpg]

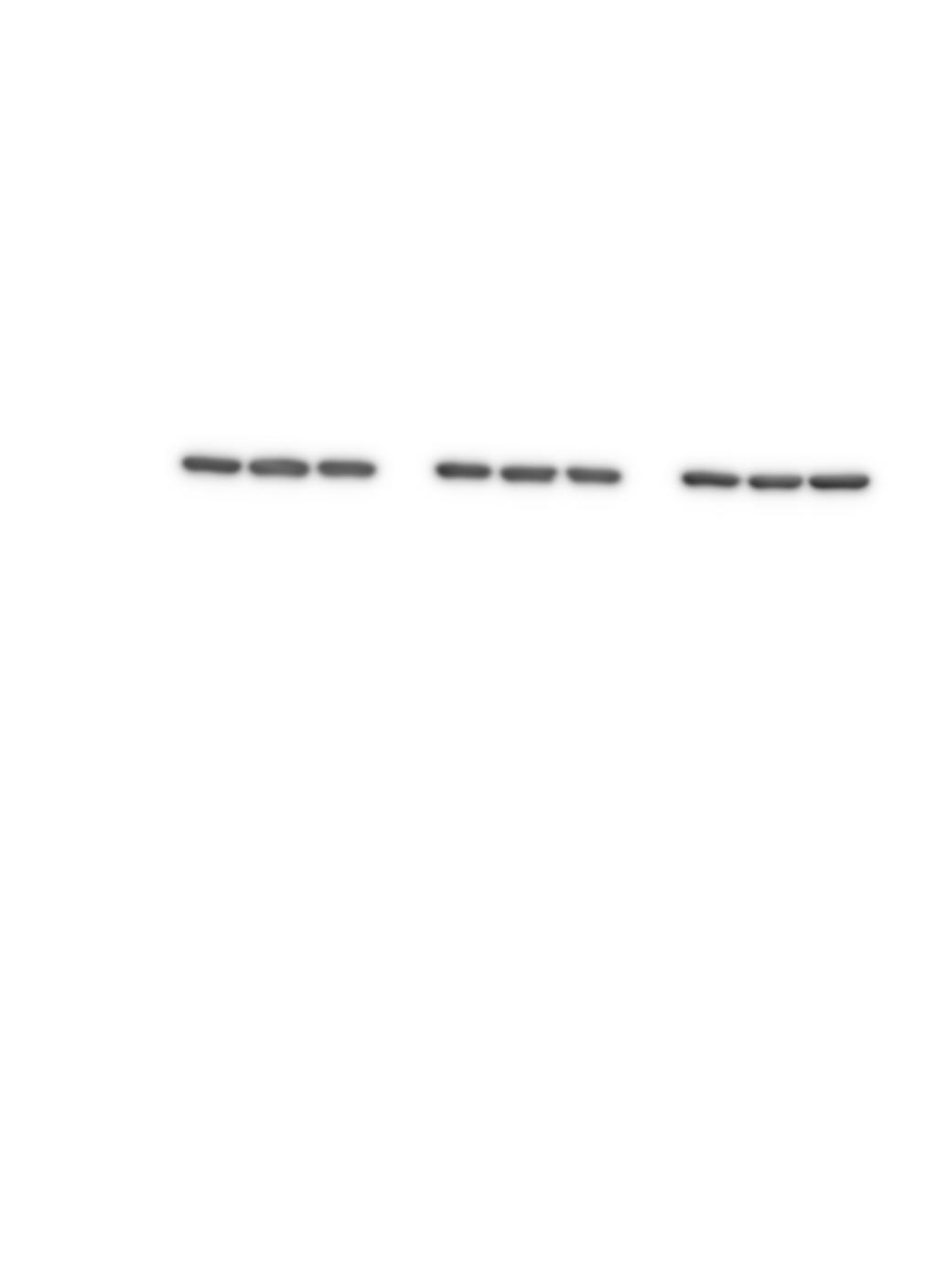

Supplement: Figure 4—figure supplement 1—source data 3. [file elife-87316-fig4-figsupp1-data3.zip › Actin_HC2022044 45 46.jpg]

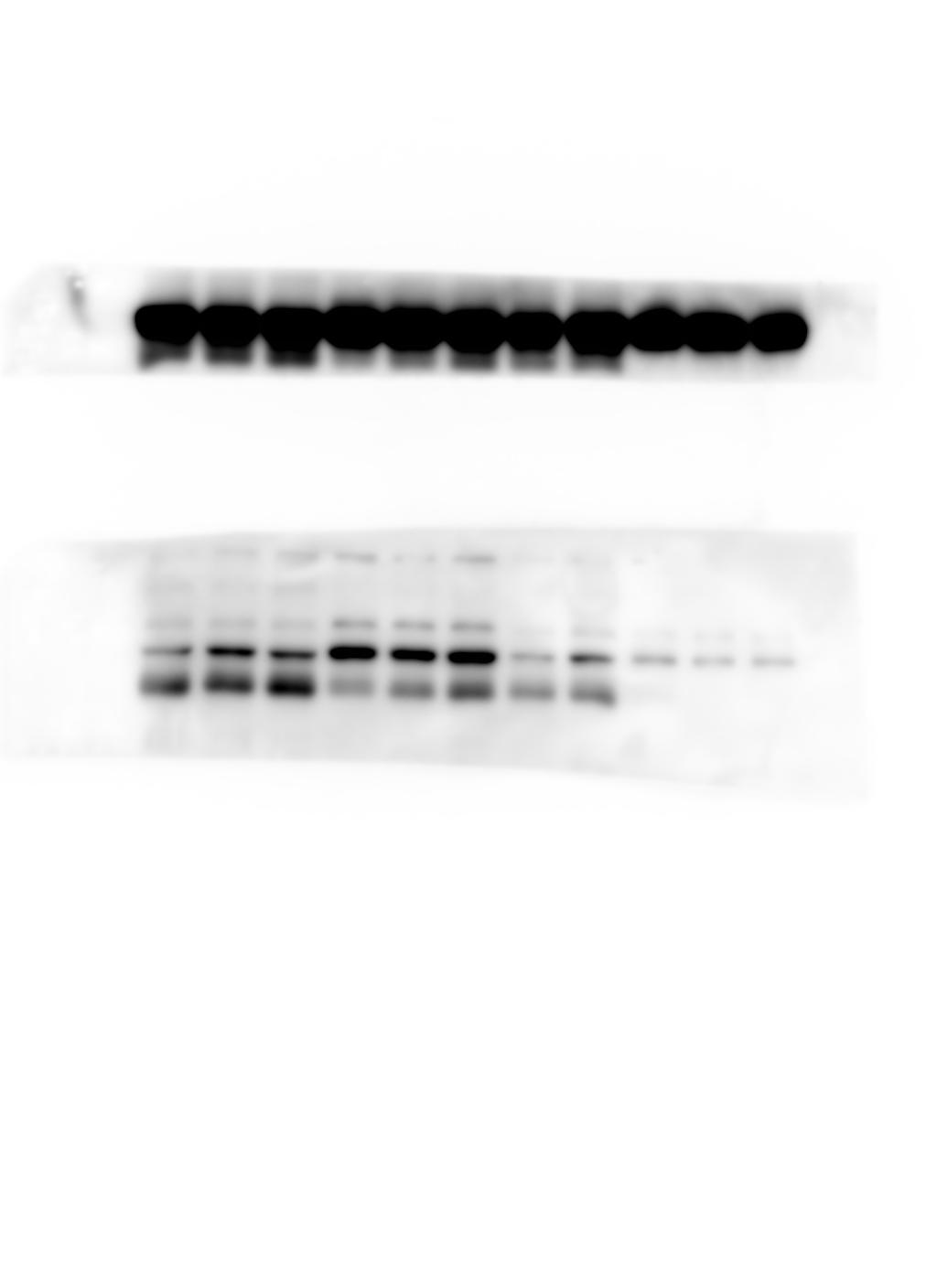

Supplement: Figure 4—figure supplement 1—source data 3. [file elife-87316-fig4-figsupp1-data3.zip › pS6K_HC2022039.jpg]

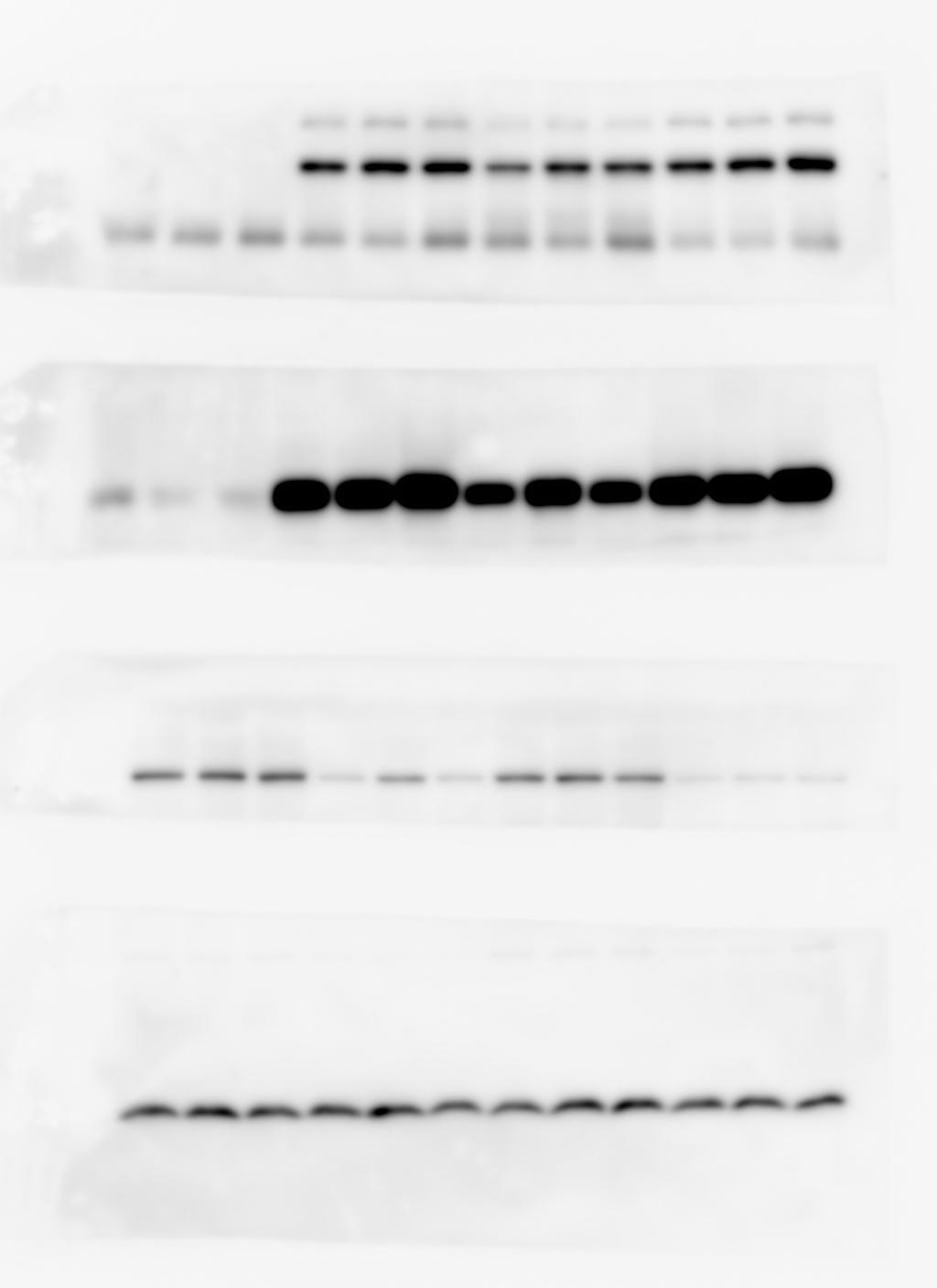

Supplement: Figure 4—figure supplement 1—source data 3. [file elife-87316-fig4-figsupp1-data3.zip › pS6K_HC2022043.jpg]

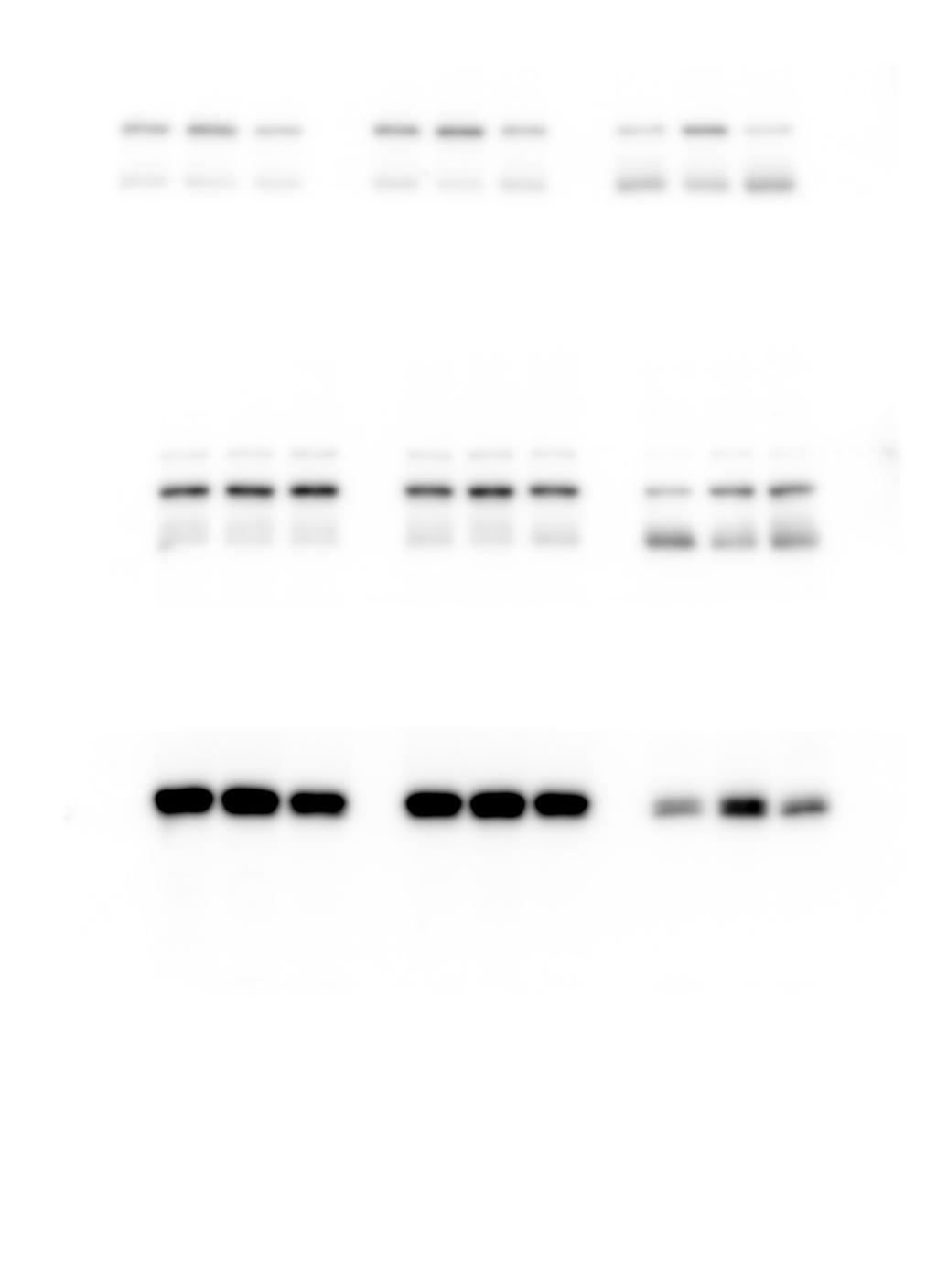

Supplement: Figure 4—figure supplement 1—source data 3. [file elife-87316-fig4-figsupp1-data3.zip › pS6K_HC2022044 45 46.jpg]

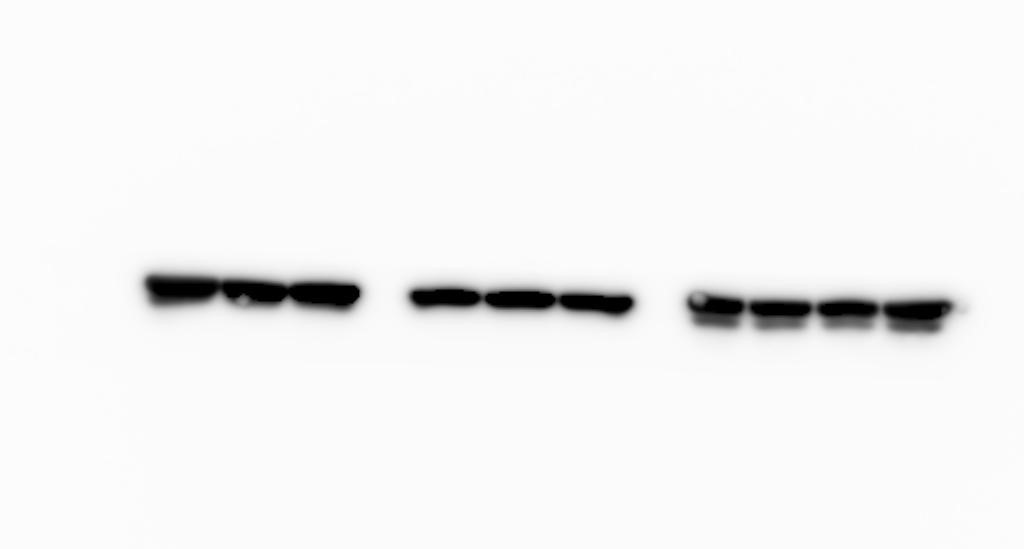

Supplement: Figure 5—source data 3. [file elife-87316-fig5-data3.zip › Actin_HC2020137.jpg]

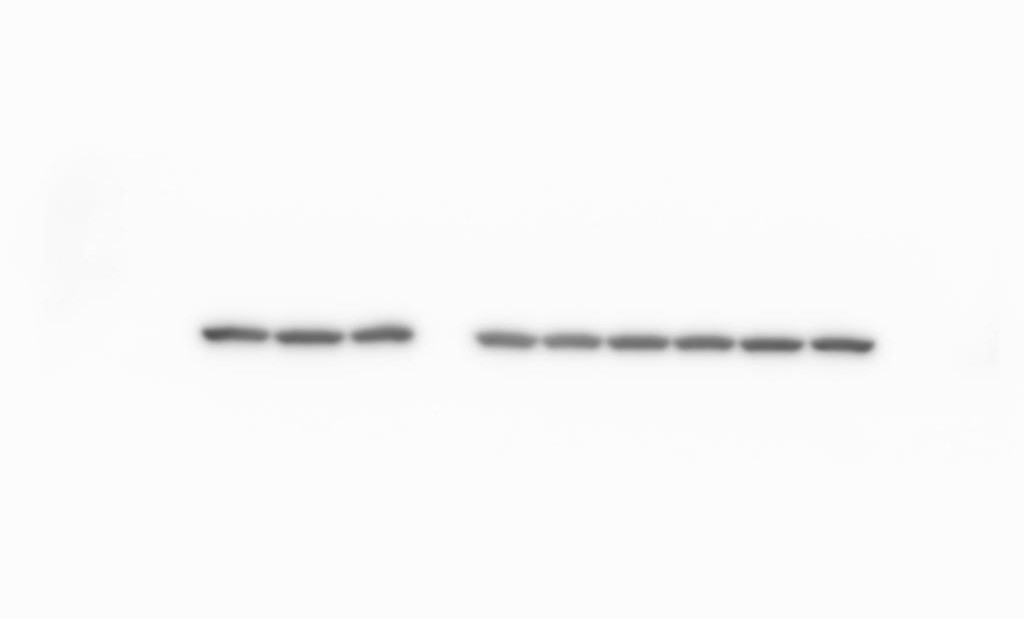

Supplement: Figure 5—source data 3. [file elife-87316-fig5-data3.zip › Actin_HC2020150.jpg]

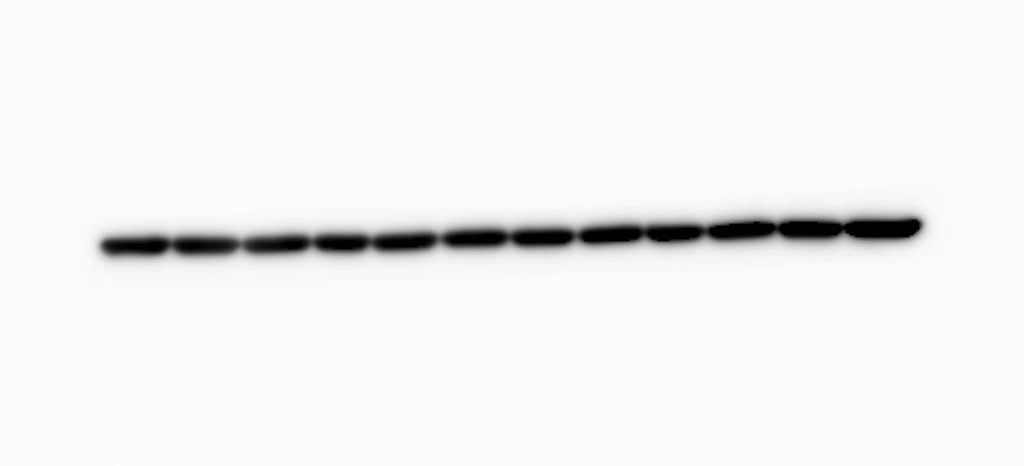

Supplement: Figure 5—source data 3. [file elife-87316-fig5-data3.zip › Actin_HC2021103.jpg]

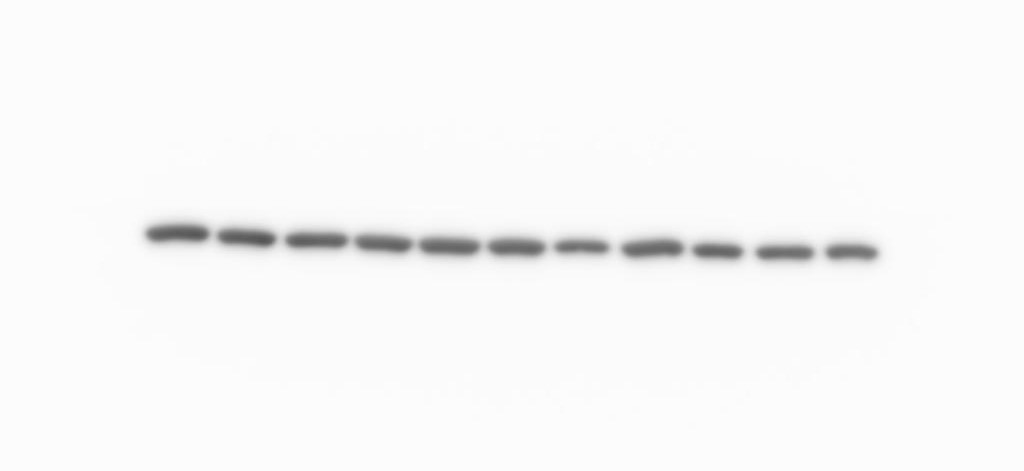

Supplement: Figure 5—source data 3. [file elife-87316-fig5-data3.zip › Actin_HC2021109.jpg]

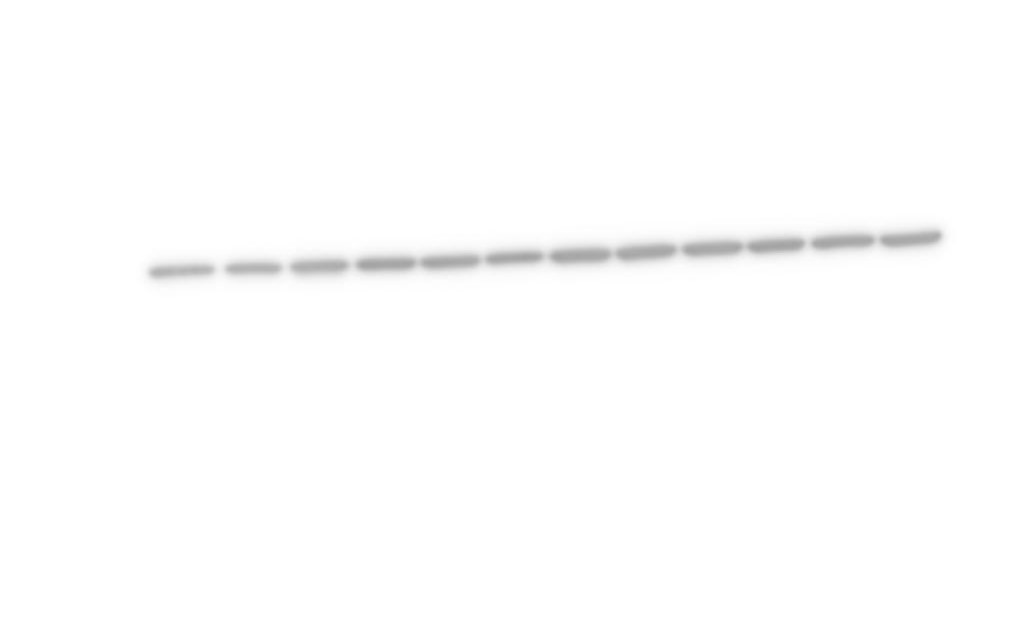

Supplement: Figure 5—source data 3. [file elife-87316-fig5-data3.zip › Actin_HC2022041.jpg]

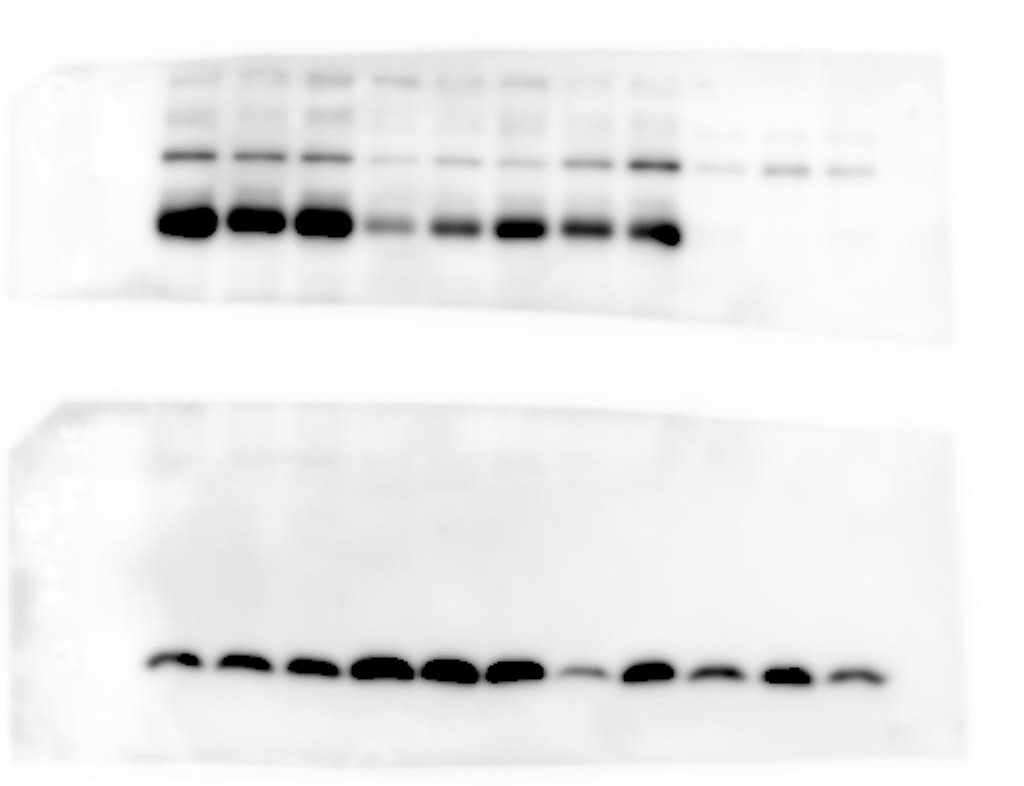

Supplement: Figure 5—source data 3. [file elife-87316-fig5-data3.zip › Alox5_Alox5ap_HC2021109.jpg]

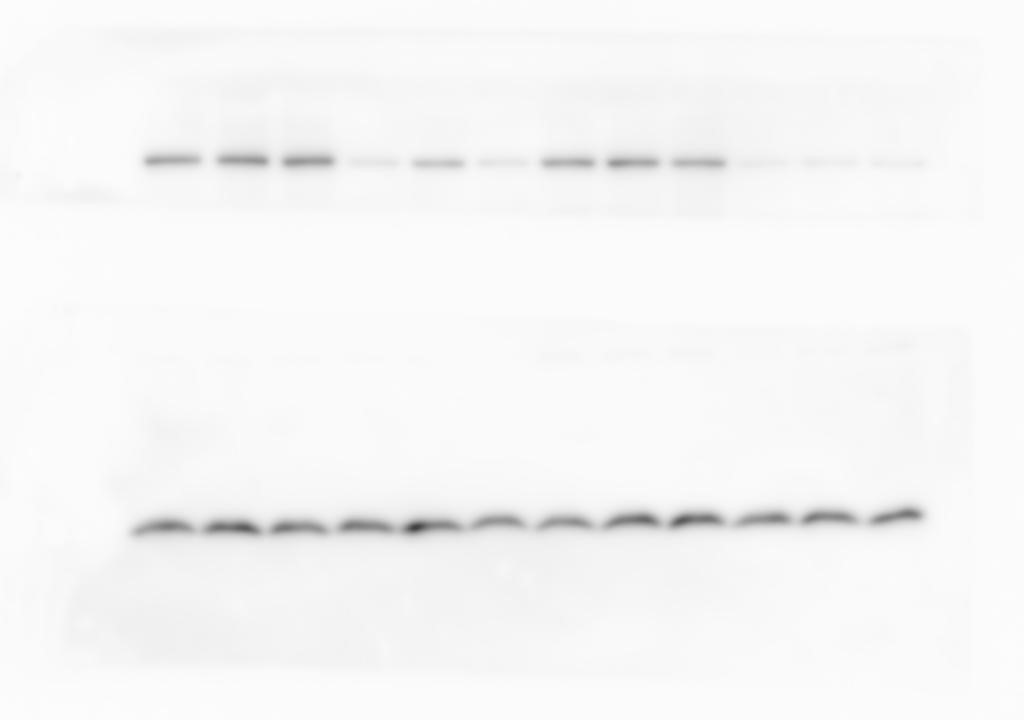

Supplement: Figure 5—source data 3. [file elife-87316-fig5-data3.zip › Alox5_Alox5ap_HC2022041.jpg]

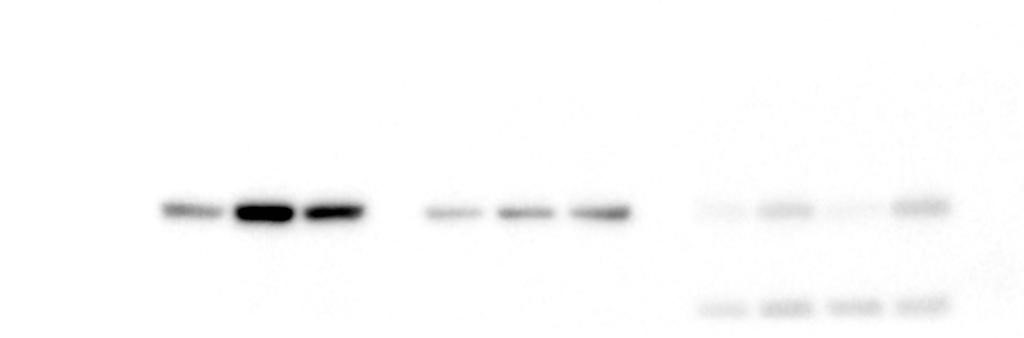

Supplement: Figure 5—source data 3. [file elife-87316-fig5-data3.zip › Alox5_HC2020137.jpg]

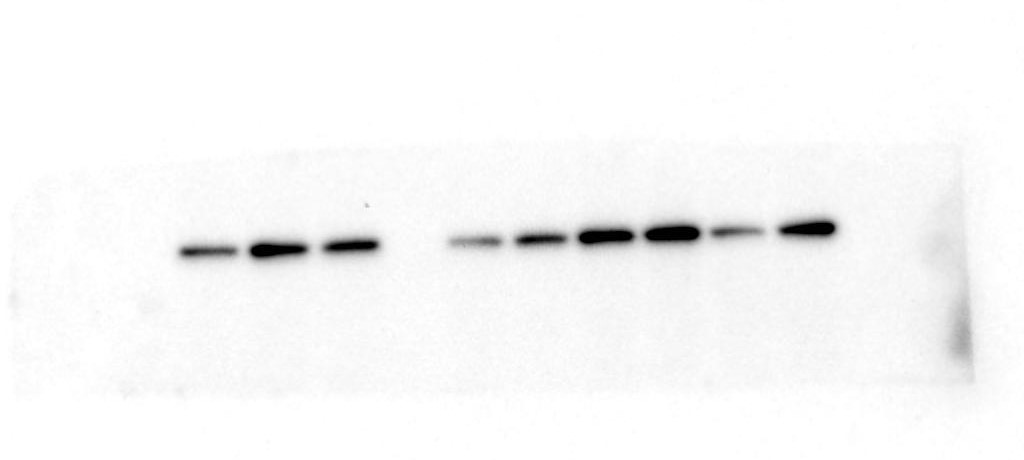

Supplement: Figure 5—source data 3. [file elife-87316-fig5-data3.zip › Alox5_HC2020150.jpg]

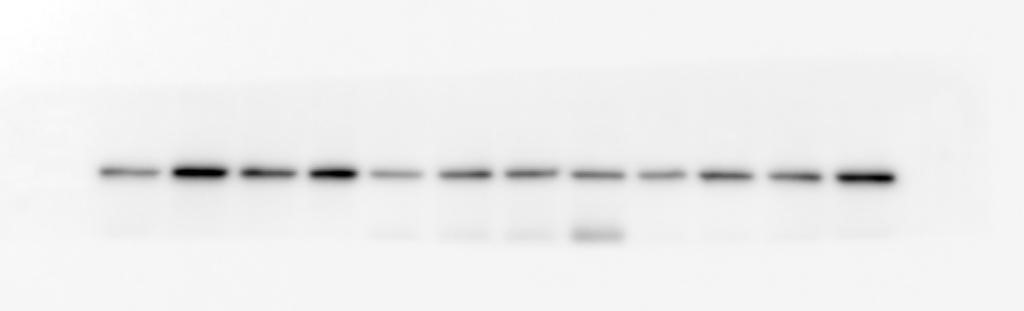

Supplement: Figure 5—source data 3. [file elife-87316-fig5-data3.zip › Alox5_HC2021103.jpg]

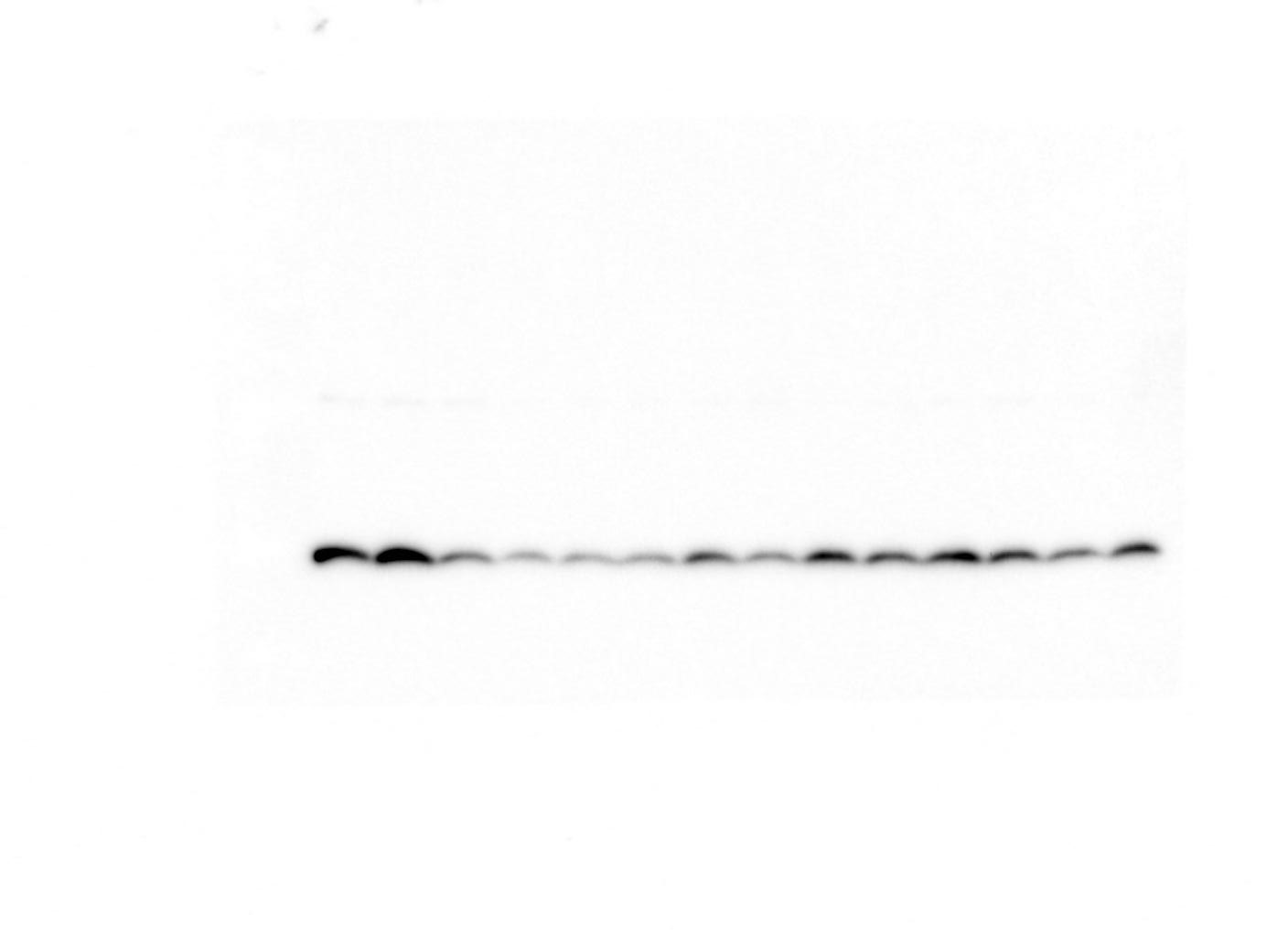

Supplement: Figure 5—source data 3. [file elife-87316-fig5-data3.zip › Alox5ap_HC2020137.jpg]

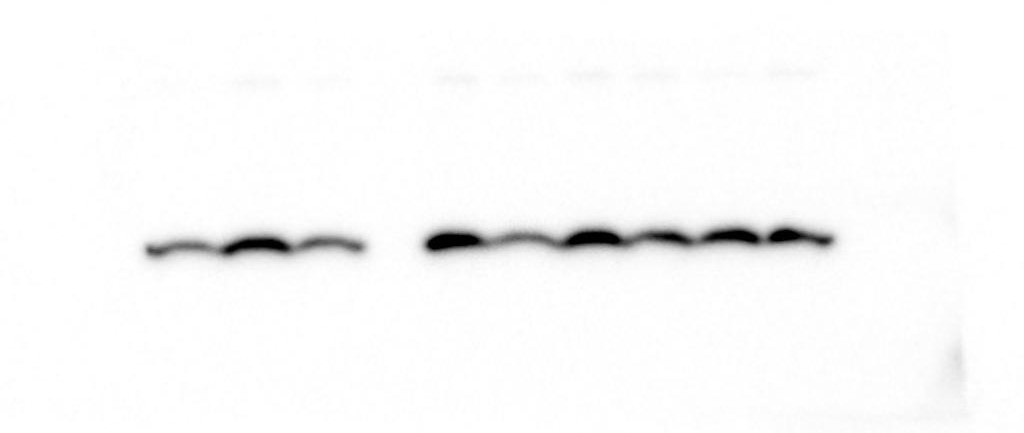

Supplement: Figure 5—source data 3. [file elife-87316-fig5-data3.zip › Alox5ap_HC2020150.jpg]

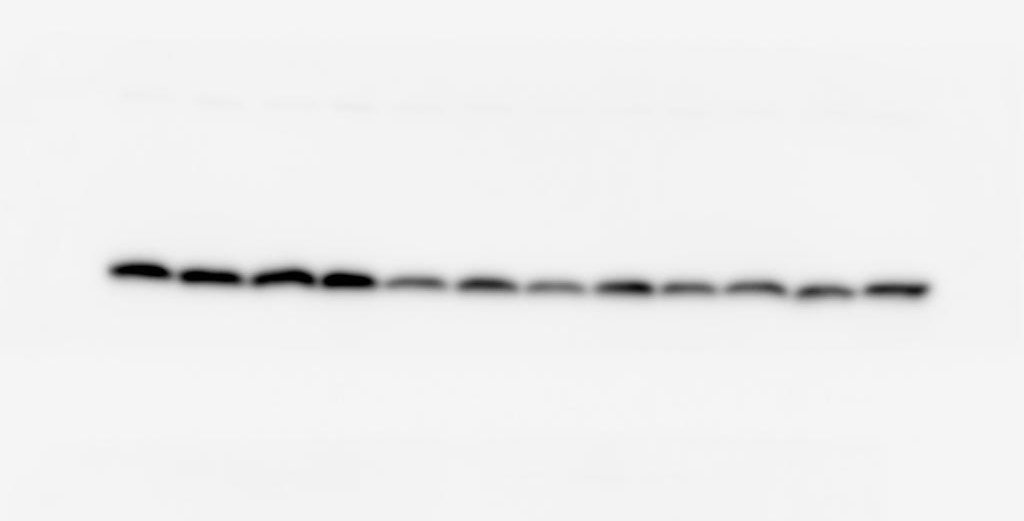

Supplement: Figure 5—source data 3. [file elife-87316-fig5-data3.zip › Alox5ap_HC2021103.jpg]

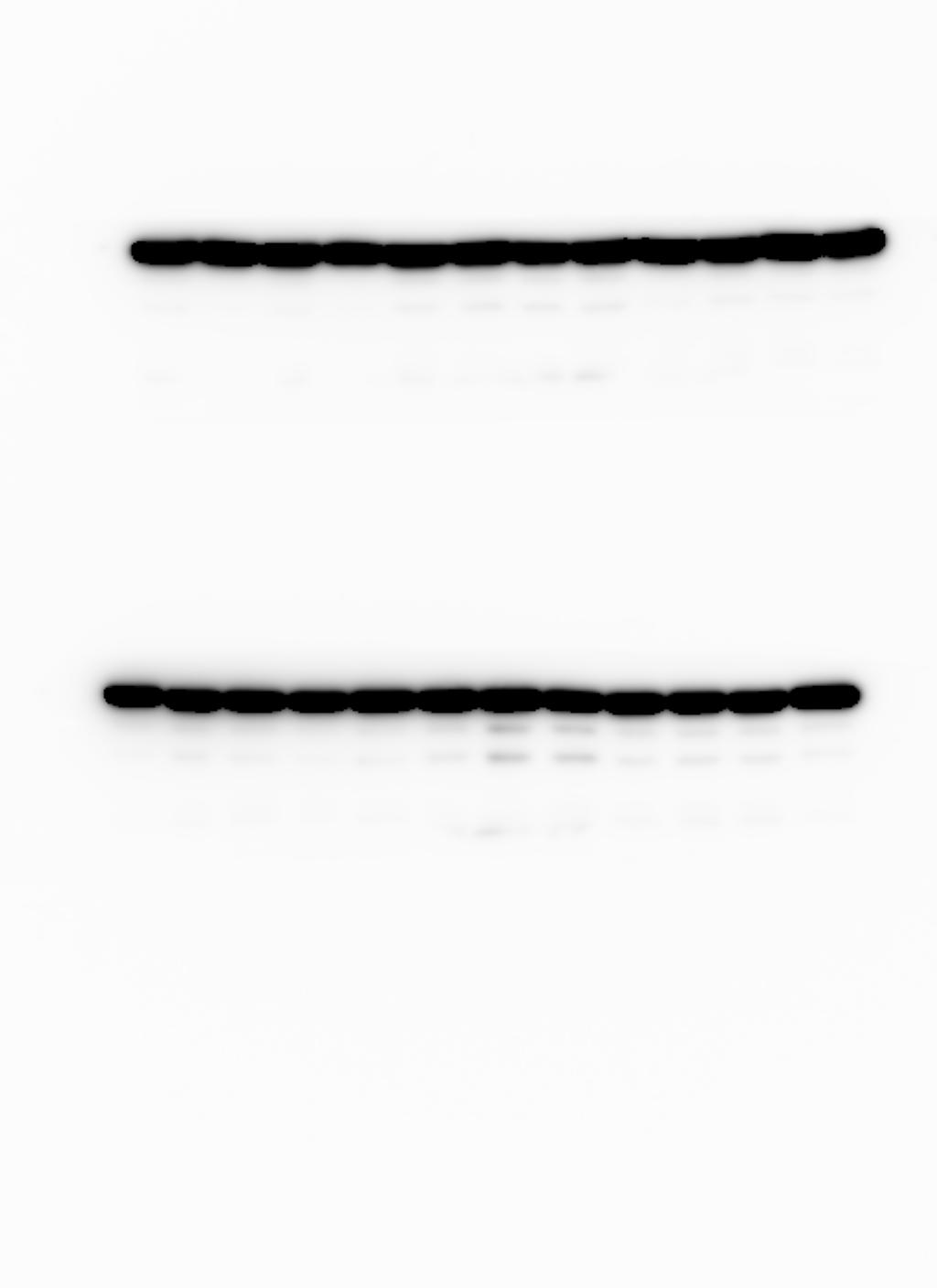

Supplement: Figure 5—figure supplement 1—source data 3. [file elife-87316-fig5-figsupp1-data3.zip › Actin_1-2.jpg]

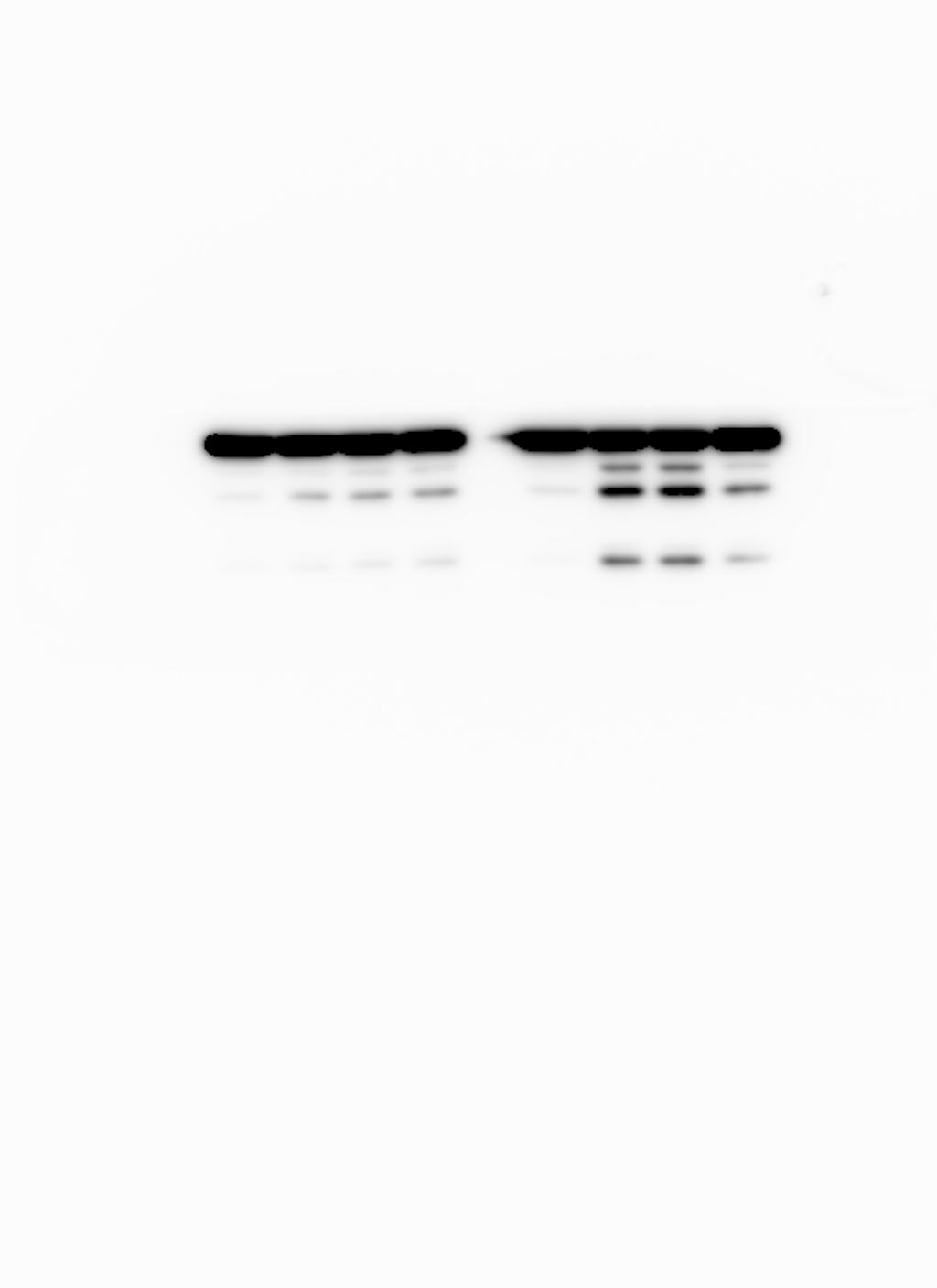

Supplement: Figure 5—figure supplement 1—source data 3. [file elife-87316-fig5-figsupp1-data3.zip › Actin_3-4.jpg]

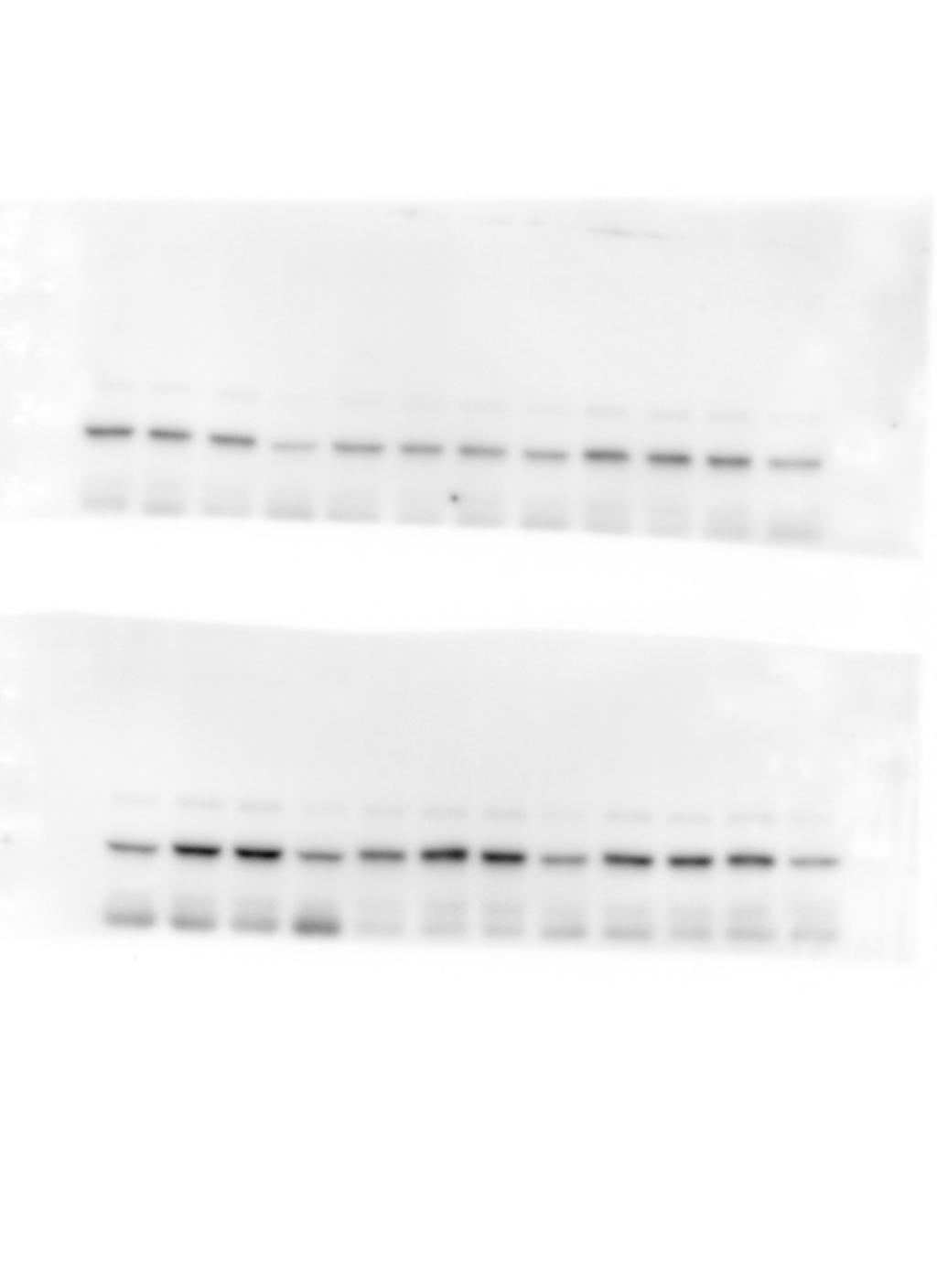

Supplement: Figure 5—figure supplement 1—source data 3. [file elife-87316-fig5-figsupp1-data3.zip › pS6K_1-2.jpg]

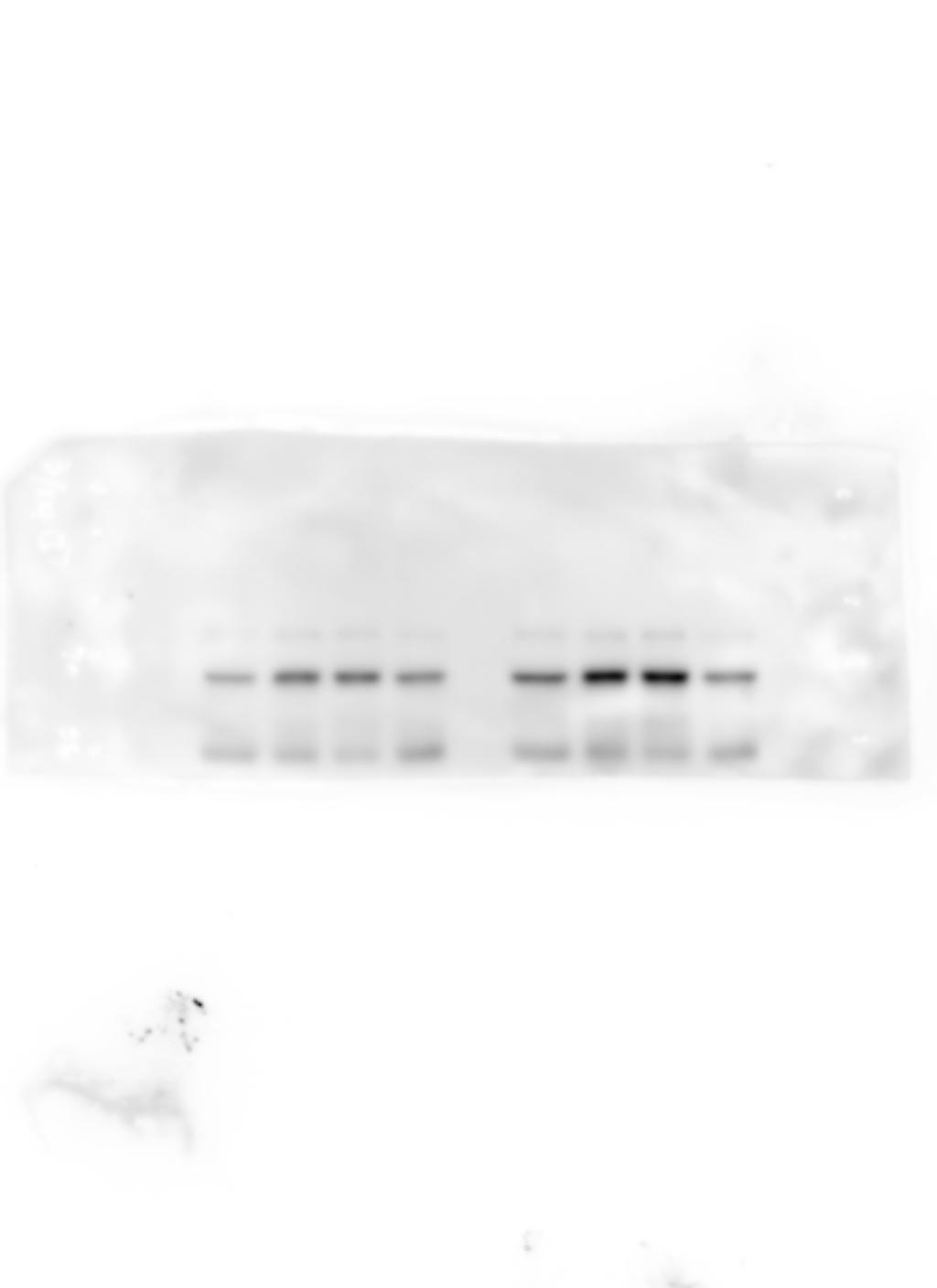

Supplement: Figure 5—figure supplement 1—source data 3. [file elife-87316-fig5-figsupp1-data3.zip › pS6K_3-4.jpg]

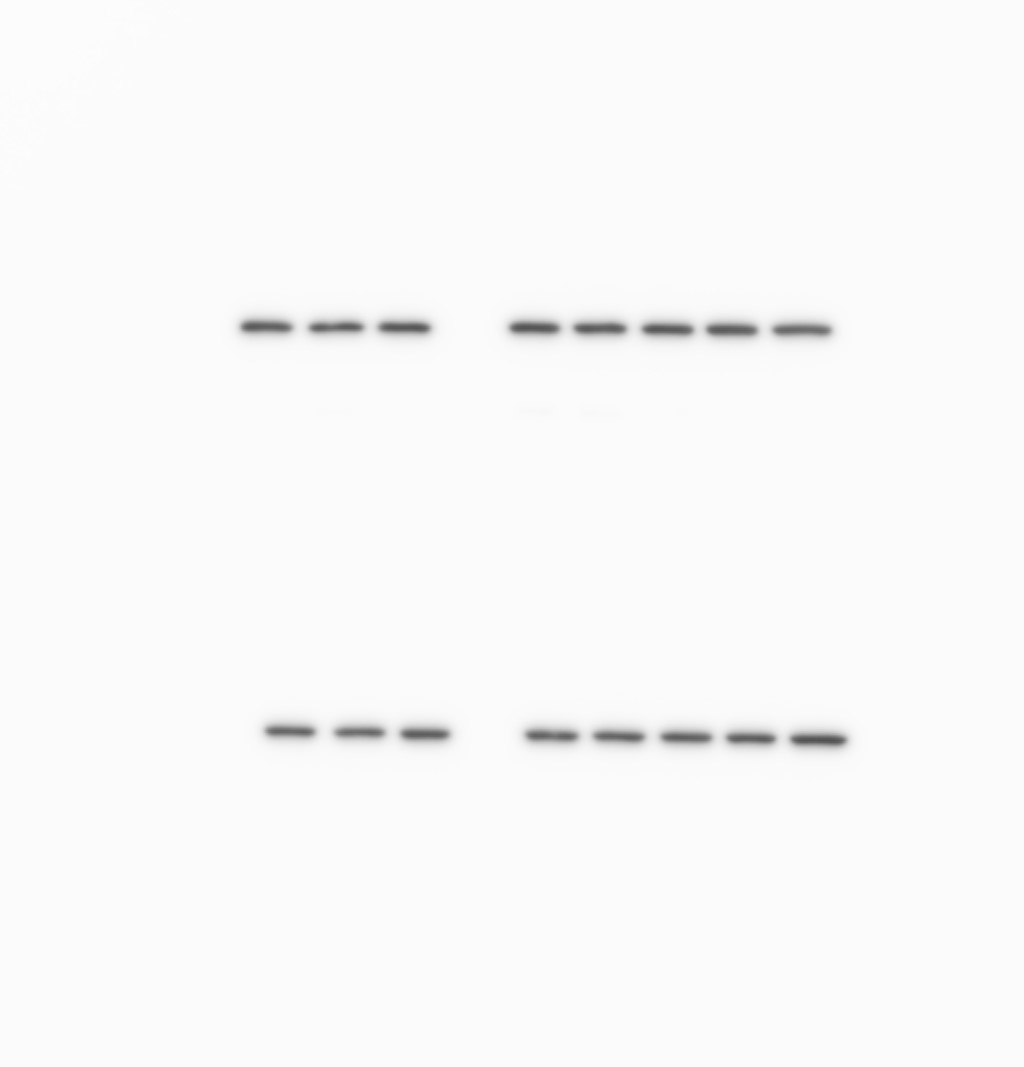

Supplement: Figure 6—source data 3. [file elife-87316-fig6-data3.zip › Figure 6E/Actin_mo.jpg]

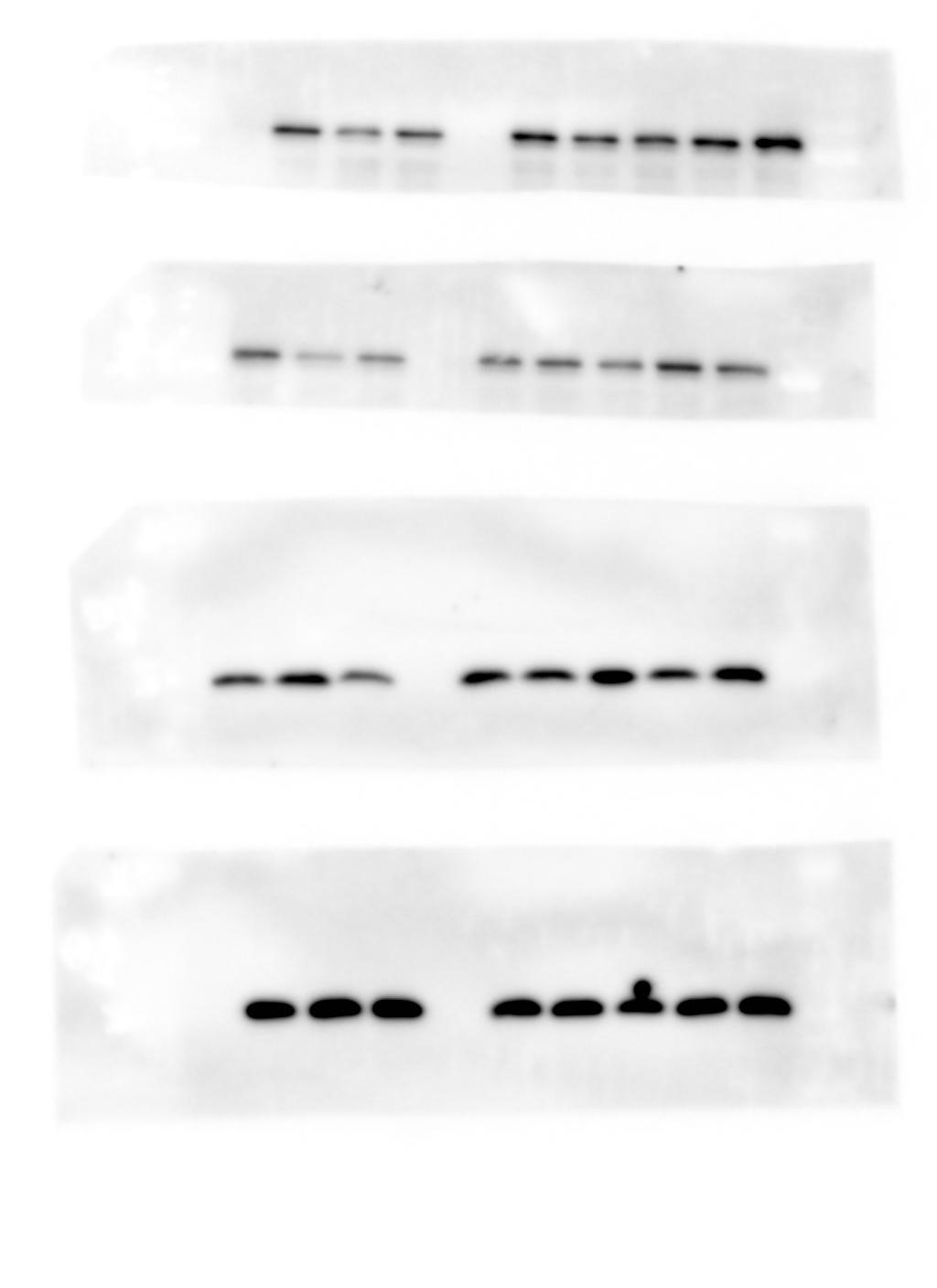

Supplement: Figure 6—source data 3. [file elife-87316-fig6-data3.zip › Figure 6E/Alox5_Alox5ap_mo.jpg]

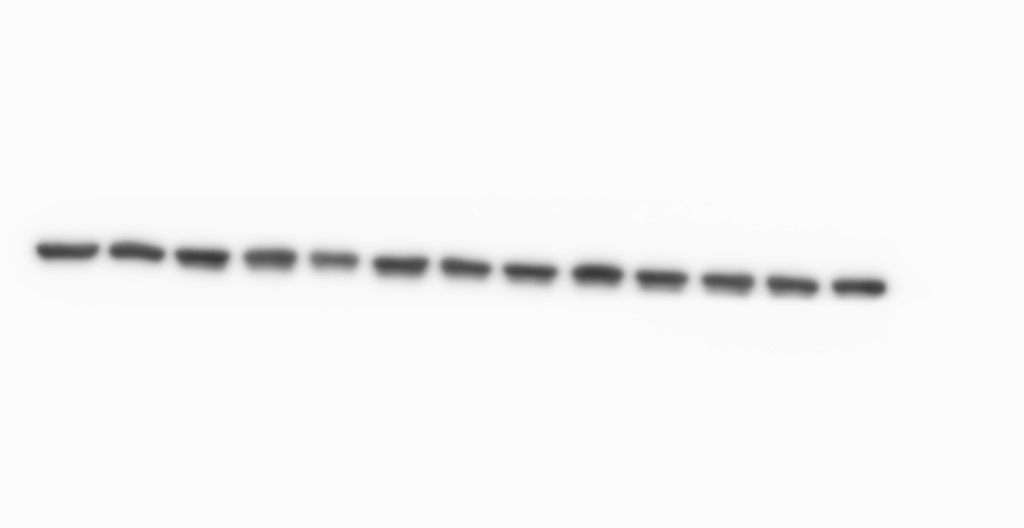

Supplement: Figure 6—source data 3. [file elife-87316-fig6-data3.zip › Figure 6G/Actin_HMDM.jpg]

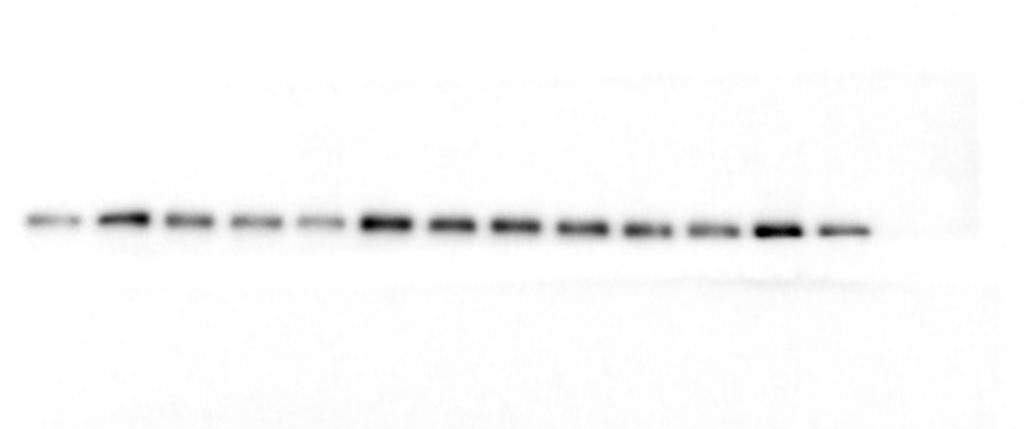

Supplement: Figure 6—source data 3. [file elife-87316-fig6-data3.zip › Figure 6G/ALOX5_HMDM.jpg]

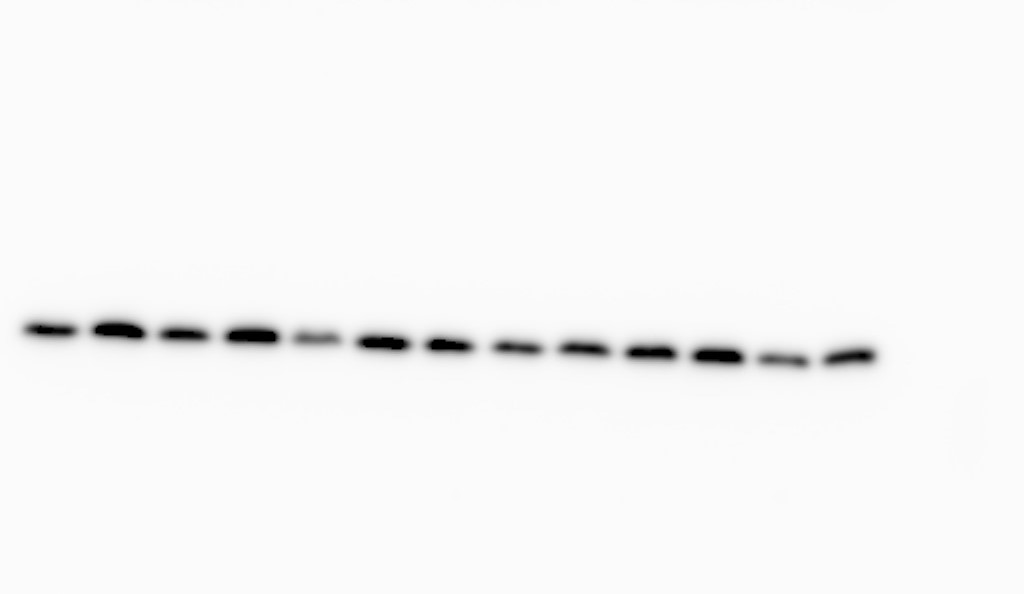

Supplement: Figure 6—source data 3. [file elife-87316-fig6-data3.zip › Figure 6G/ALOX5AP_HMDM.jpg]

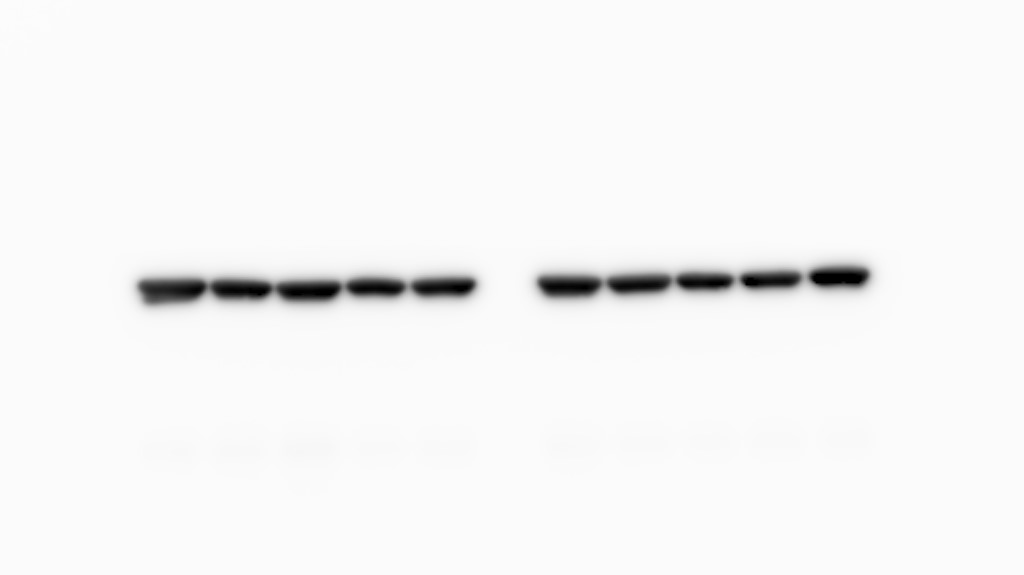

Supplement: Figure 6—source data 3. [file elife-87316-fig6-data3.zip › Figure 6L/mActin_sp.jpg]

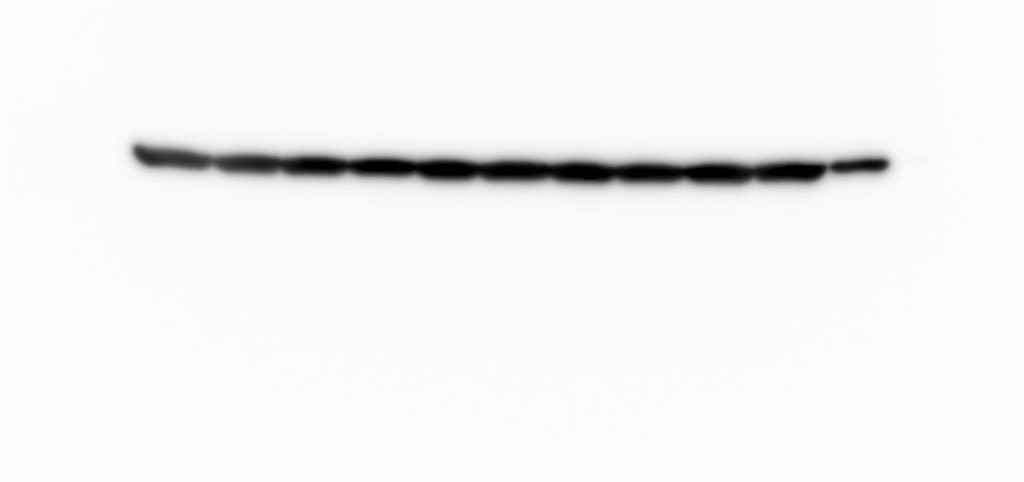

Supplement: Figure 6—source data 3. [file elife-87316-fig6-data3.zip › Figure 6L/mActin-1_sp.jpg]

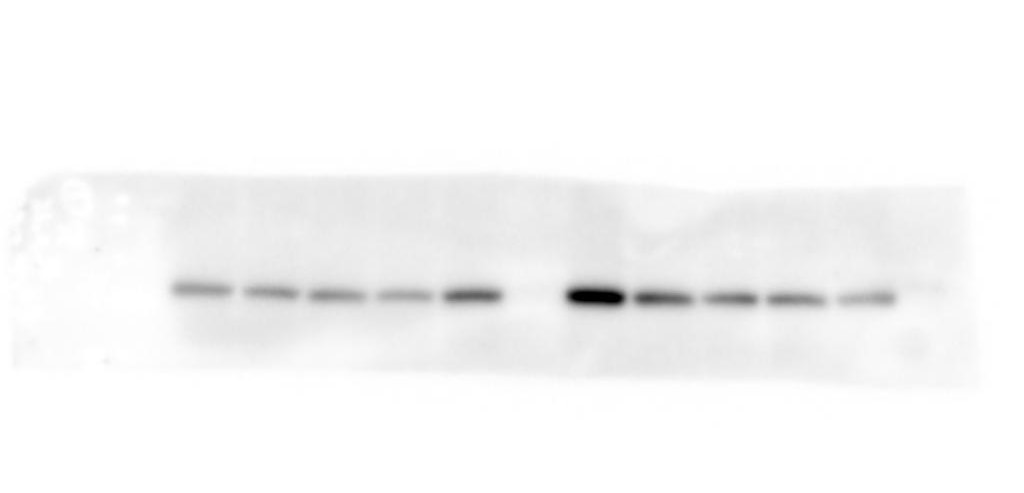

Supplement: Figure 6—source data 3. [file elife-87316-fig6-data3.zip › Figure 6L/mALOX5_sp.jpg]

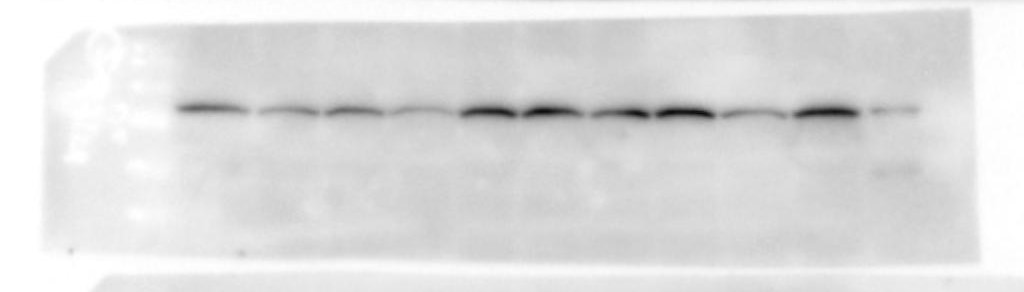

Supplement: Figure 6—source data 3. [file elife-87316-fig6-data3.zip › Figure 6L/mALOX5-1_sp.jpg]

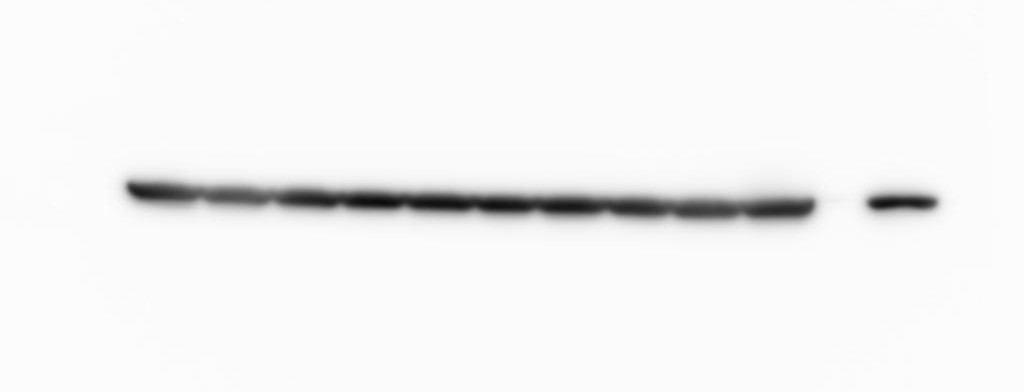

Supplement: Figure 6—figure supplement 1—source data 3. [file elife-87316-fig6-figsupp1-data3.zip › Actin_BM.jpg]

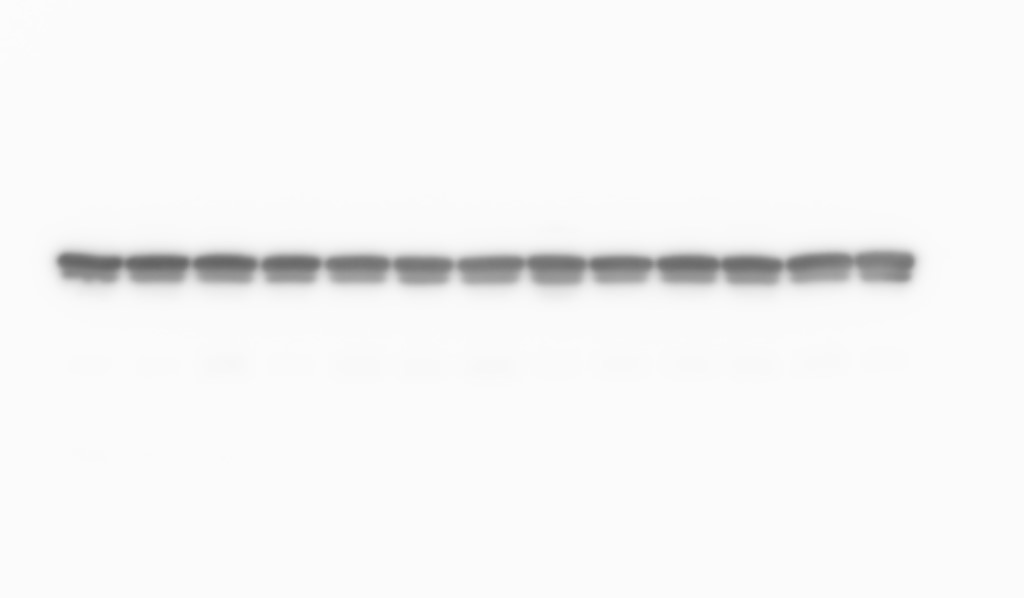

Supplement: Figure 6—figure supplement 1—source data 3. [file elife-87316-fig6-figsupp1-data3.zip › Actin_BM-1.jpg]

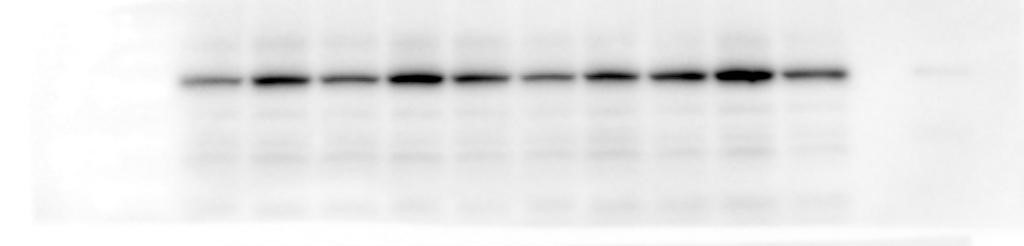

Supplement: Figure 6—figure supplement 1—source data 3. [file elife-87316-fig6-figsupp1-data3.zip › Alox5_BM.jpg]

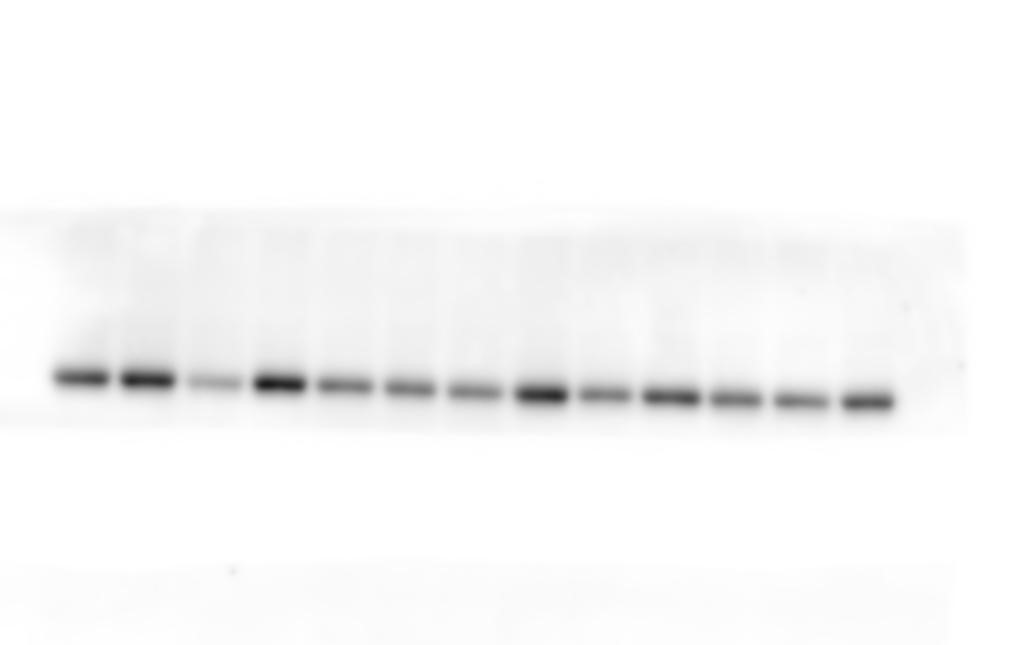

Supplement: Figure 6—figure supplement 1—source data 3. [file elife-87316-fig6-figsupp1-data3.zip › Alox5_BM-1.jpg]
