# Supplementary material for: Uremic toxin indoxyl sulfate induces trained immunity via the AhR-dependent arachidonic acid pathway in end-stage renal disease (ESRD)
Source: eLife. 2024 Jul 9;12:RP87316. doi: 10.7554/eLife.87316 (PMC11233136; doi:10.7554/eLife.87316)

Figure 4-figure supplement 1D, western blotting data

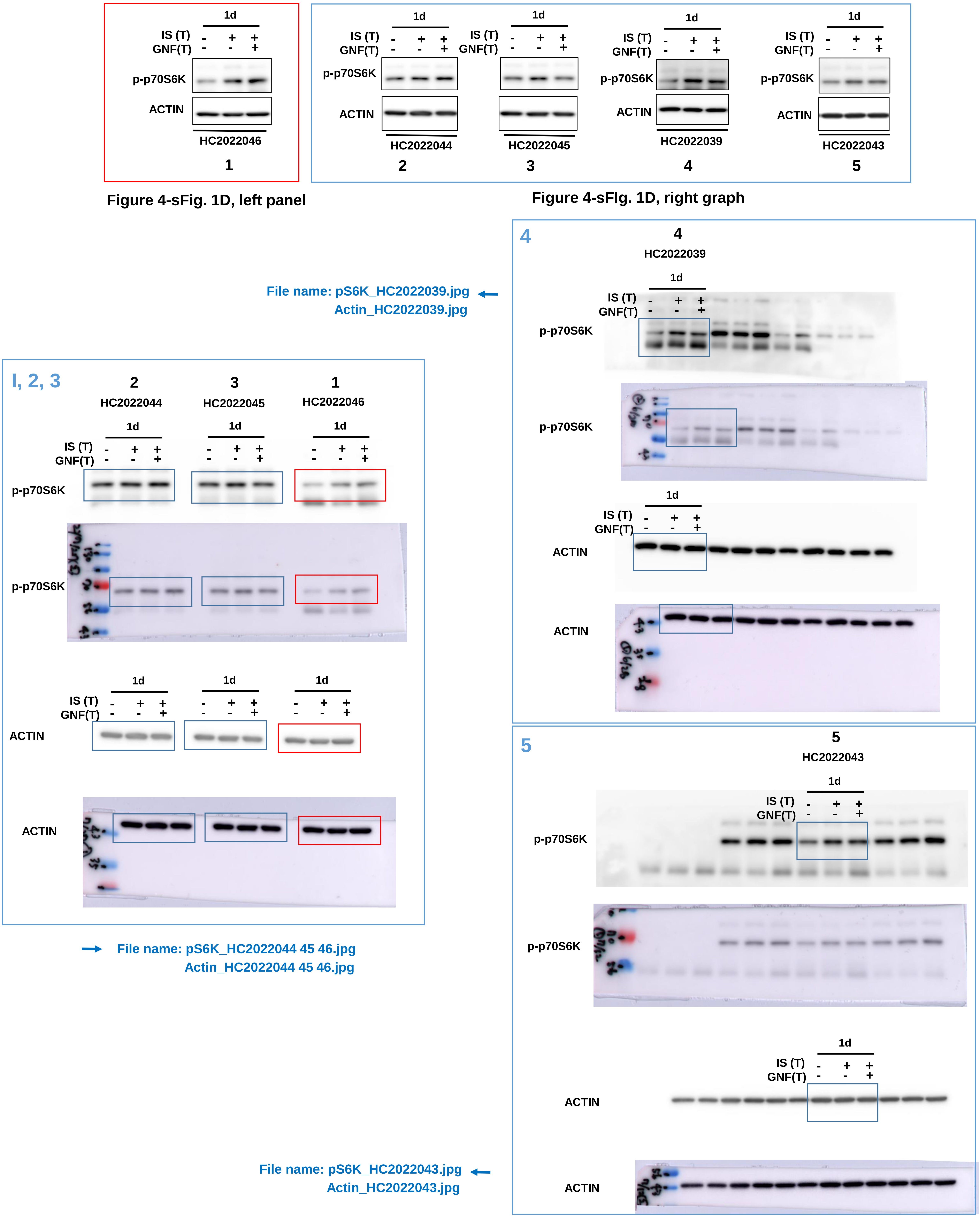

Supplement: Figure 4—figure supplement 1—source data 2. [file elife-87316-fig4-figsupp1-data2.pdf]
