# Supplementary material for: Uremic toxin indoxyl sulfate induces trained immunity via the AhR-dependent arachidonic acid pathway in end-stage renal disease (ESRD)
Source: eLife. 2024 Jul 9;12:RP87316. doi: 10.7554/eLife.87316 (PMC11233136; doi:10.7554/eLife.87316)
Supplement: Figure 5—source data 2. [file elife-87316-fig5-data2.pdf]

Figure 5F, western blotting data

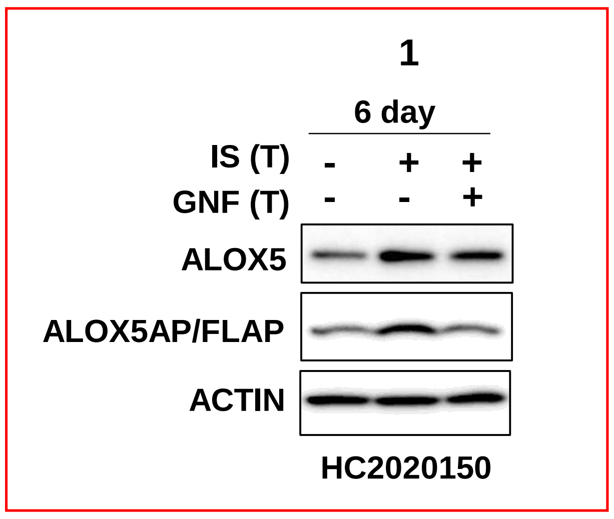

Figure 5F, left panel

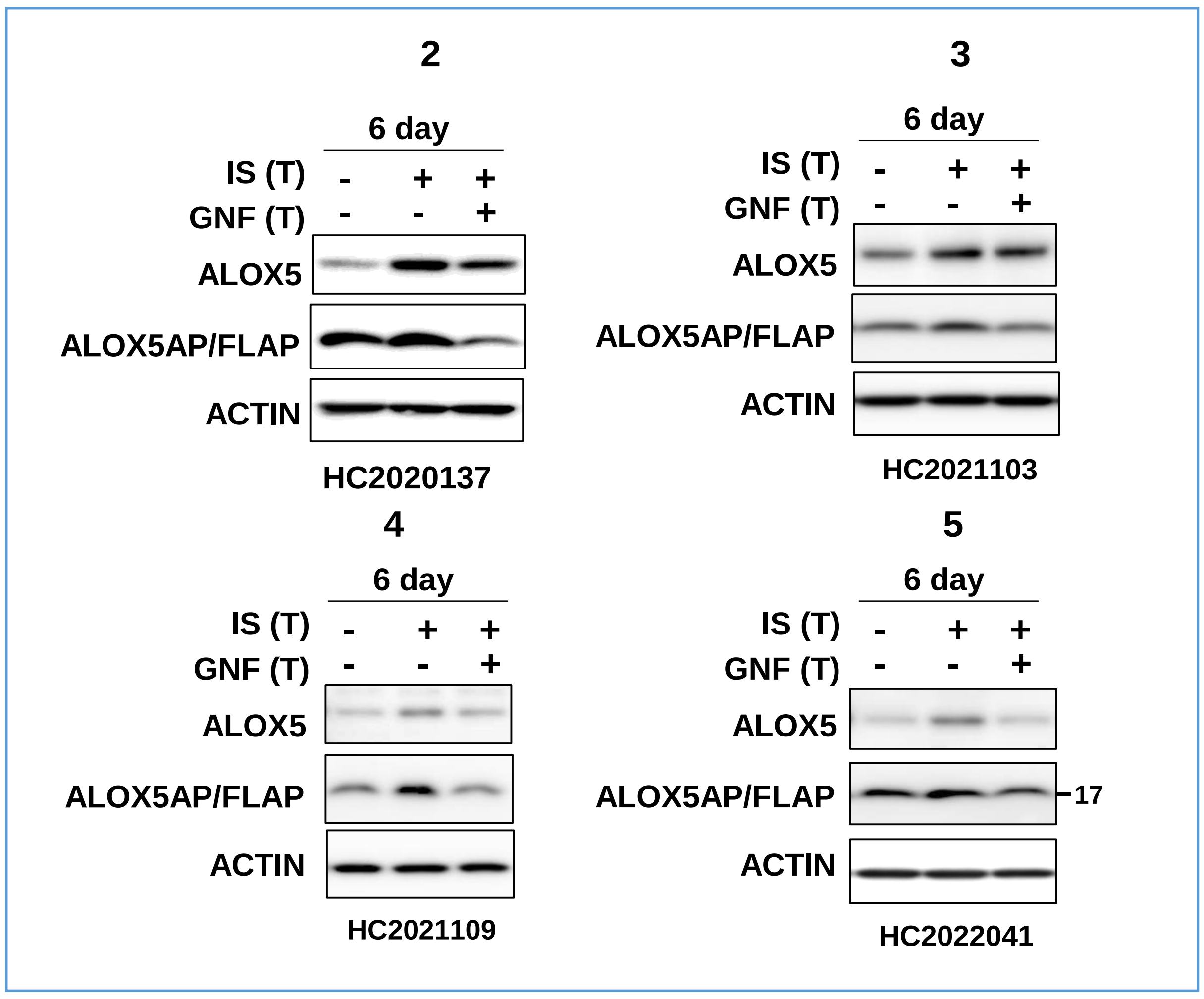

Figure 5F, right graph

Figure 5F, western blotting data

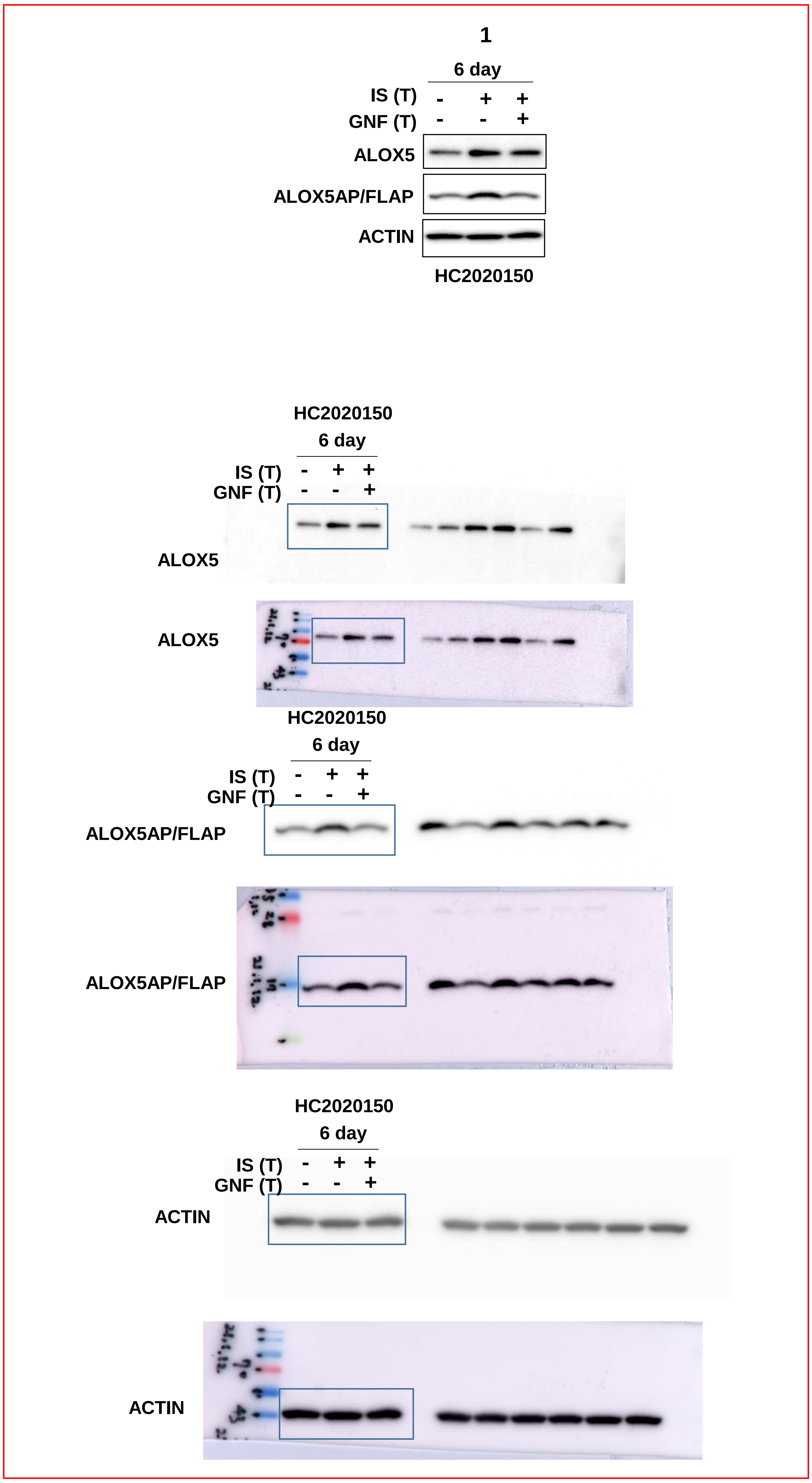

File name: Alox5_HC2020150.jpg
Alox5ap_HC2020150.jpg
Actin_HC2020150.jpg

Figure 5F, western blotting data

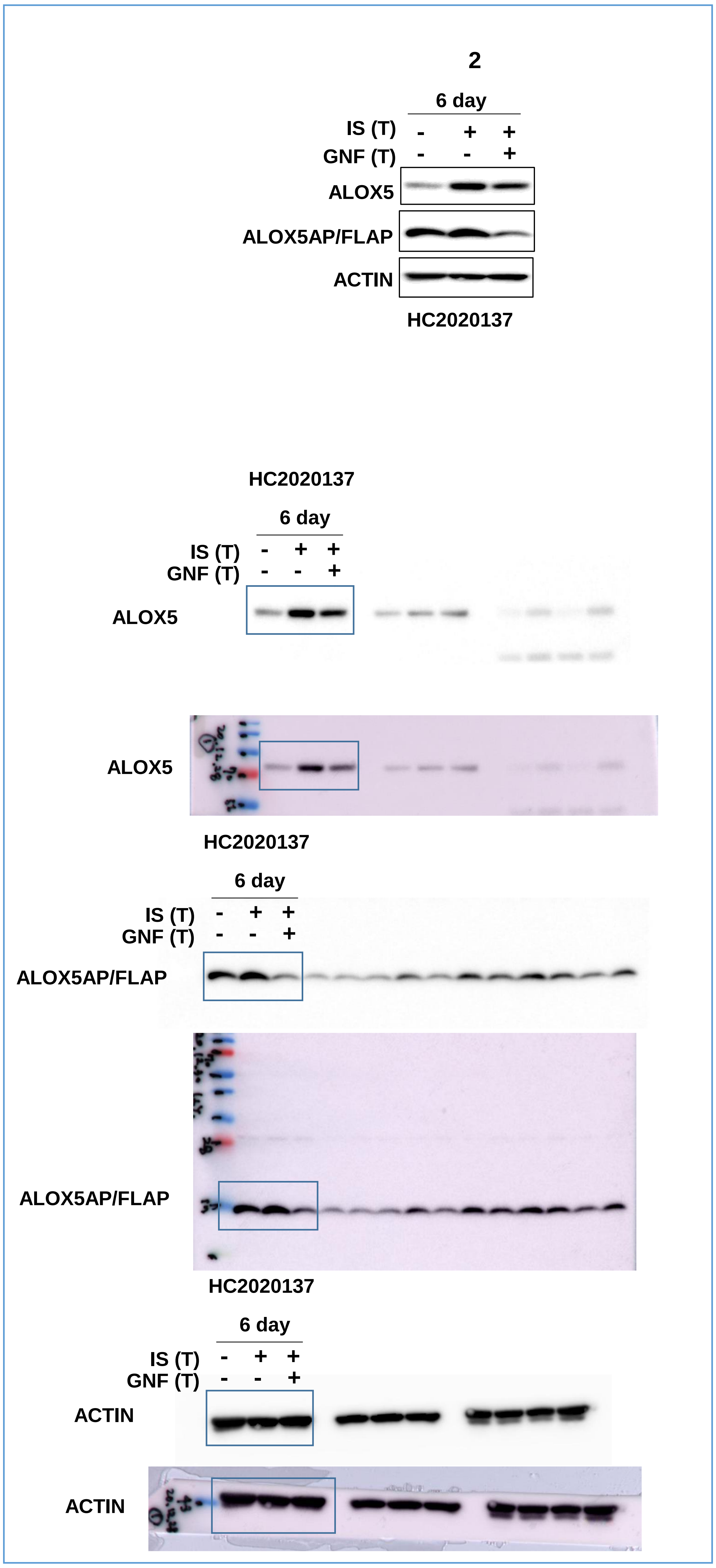

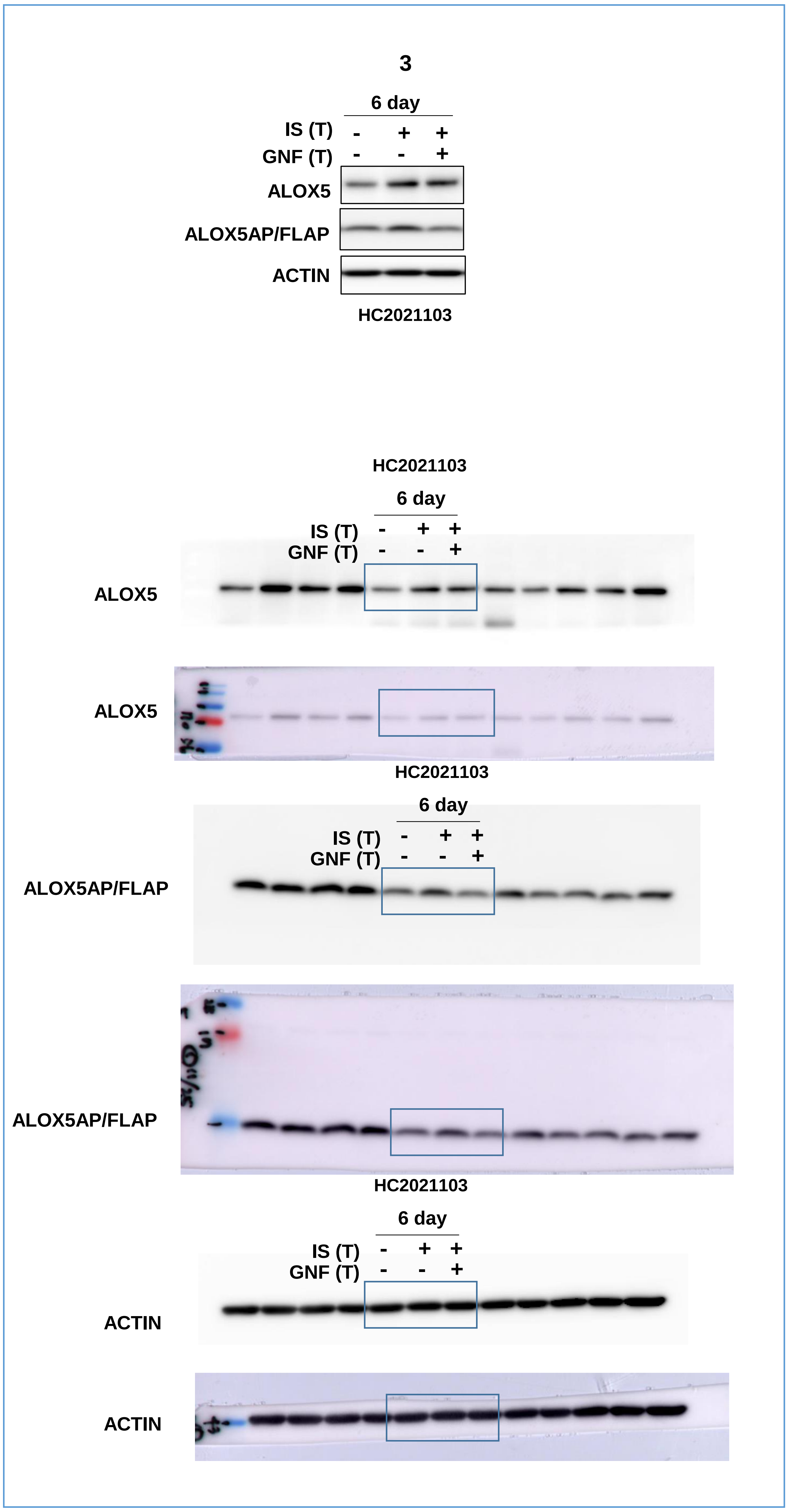

→ File name: Alox5_HC2020137.jpg
Alox5ap_HC2020137.jpg
Actin_HC2020137.jpg

→ File name: Alox5_HC2021103.jpg
Alox5ap_HC2021103.jpg
Actin_HC2021103.jpg

Figure 5F, western blotting data

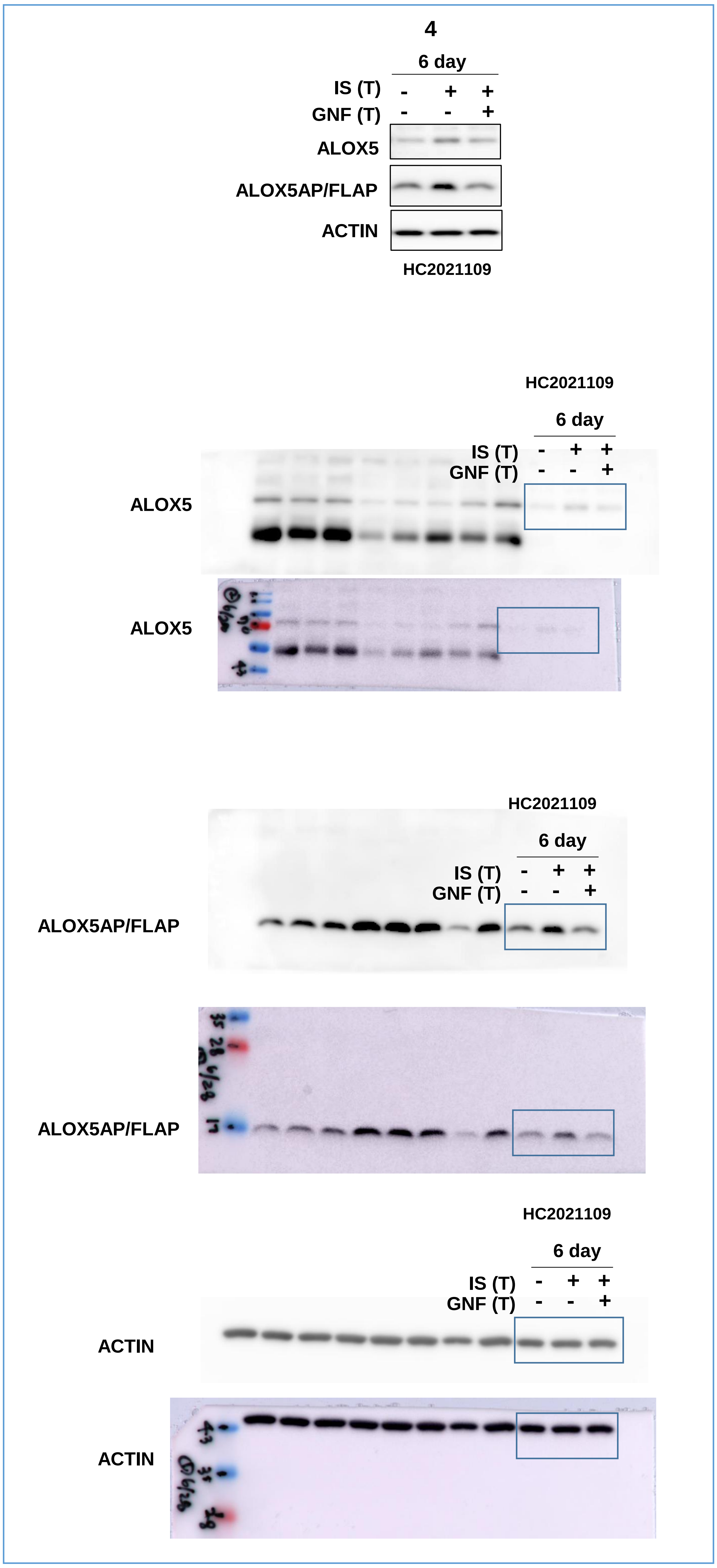

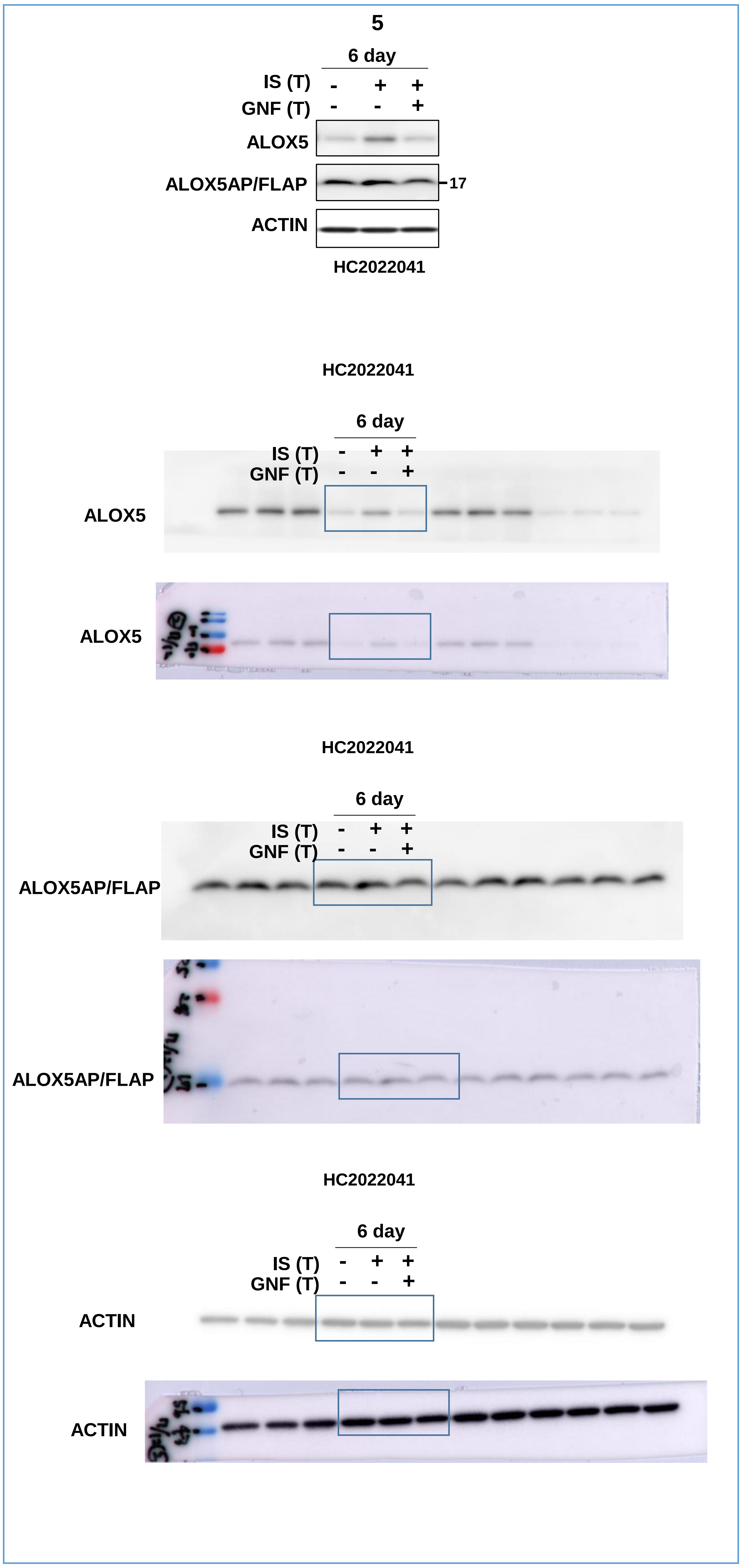

→ File name: Alox5_Alox5ap_HC2021109.jpg
Actin_HC2021109.jpg

→ File name: Alox5_Alox5ap_HC2022041.jpg
Actin_HC2022041.jpg
