# Supplementary material for: Uremic toxin indoxyl sulfate induces trained immunity via the AhR-dependent arachidonic acid pathway in end-stage renal disease (ESRD)
Source: eLife. 2024 Jul 9;12:RP87316. doi: 10.7554/eLife.87316 (PMC11233136; doi:10.7554/eLife.87316)
Supplement: Figure 5—figure supplement 1—source data 2. [file elife-87316-fig5-figsupp1-data2.pdf]

Figure 5-figure supplement 1I, western blotting data

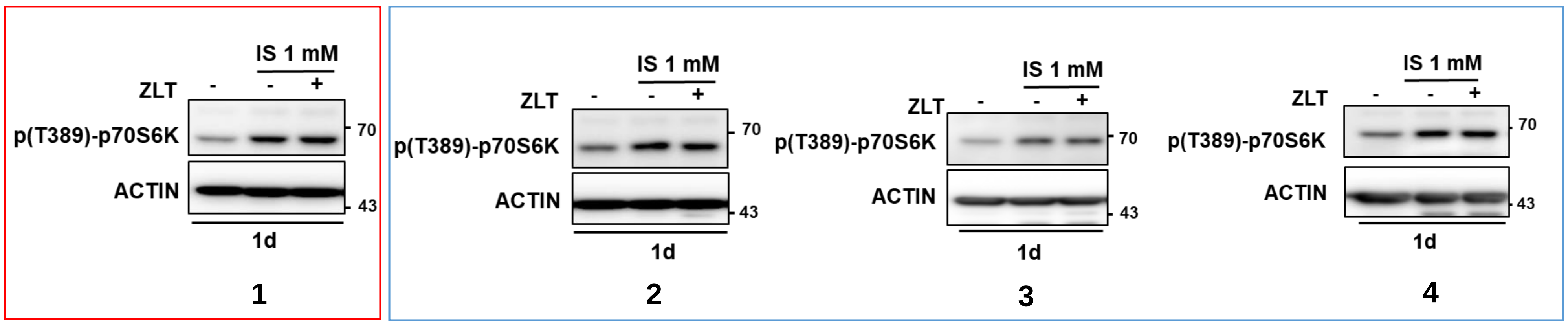

Figure 5-sFig. 1I, left panel

Figure 5-sFig. 1I, right graph

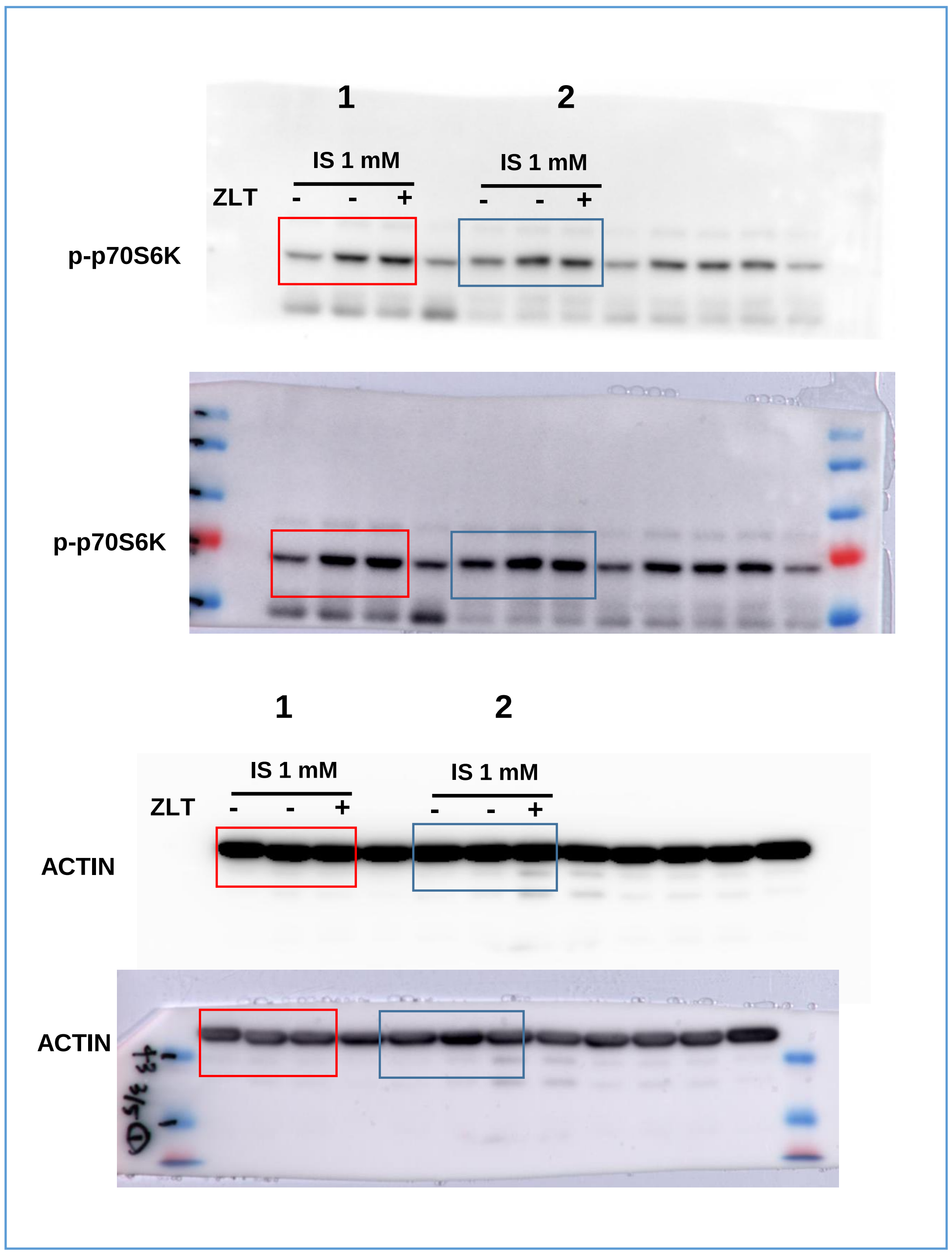

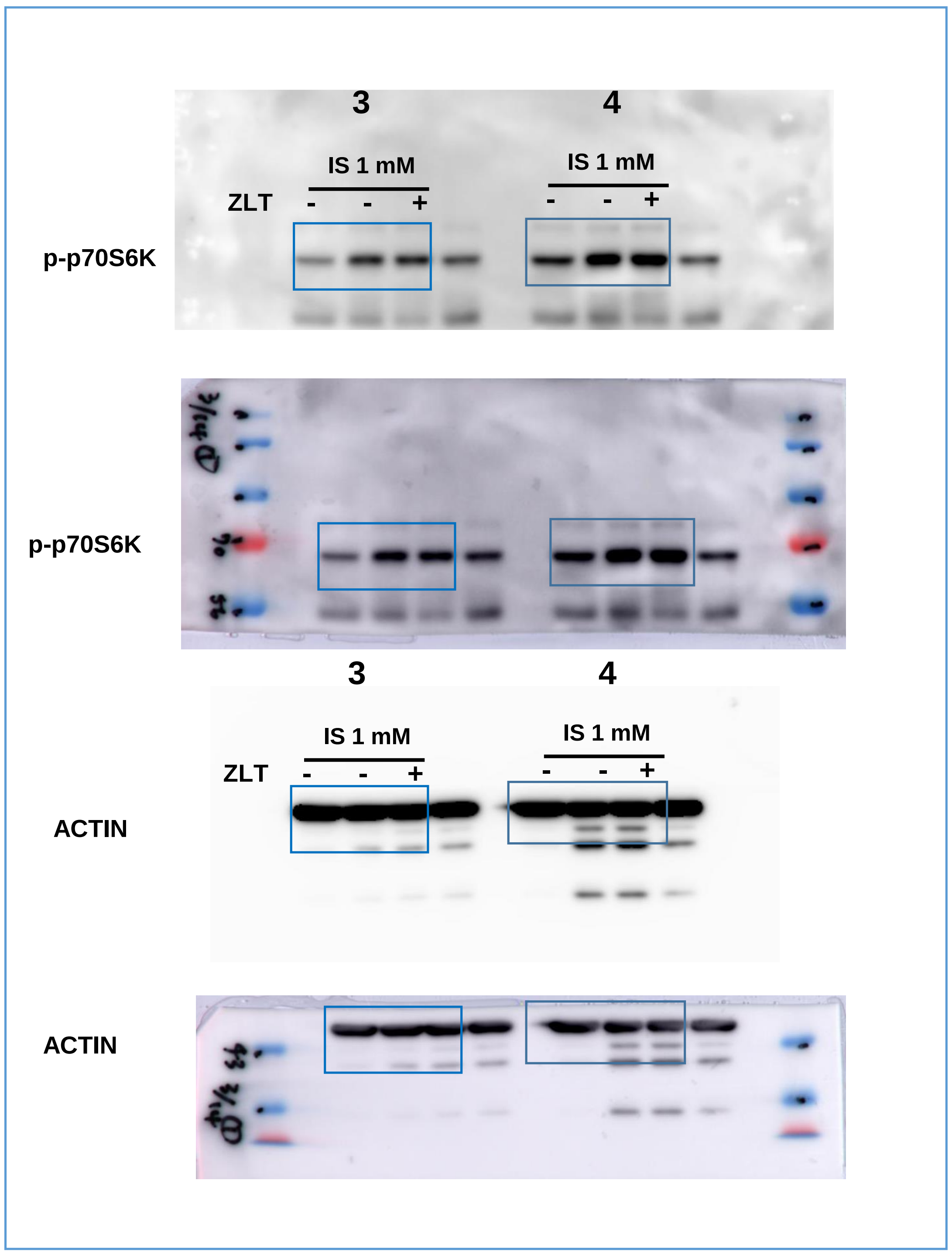

→ File name: pS6K_1-2.jpg
Actin_1-2.jpg

→ File name: pS6K_3-4.jpg
Actin_3-4.jpg
