# Supplementary material for: Uremic toxin indoxyl sulfate induces trained immunity via the AhR-dependent arachidonic acid pathway in end-stage renal disease (ESRD)
Source: eLife. 2024 Jul 9;12:RP87316. doi: 10.7554/eLife.87316 (PMC11233136; doi:10.7554/eLife.87316)
Supplement: Figure 6—source data 2. [file elife-87316-fig6-data2.pdf]

Figure 6E, western blotting data

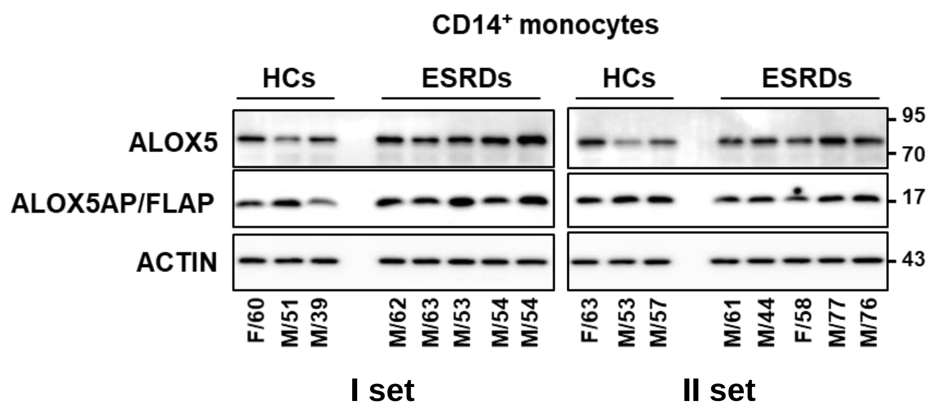

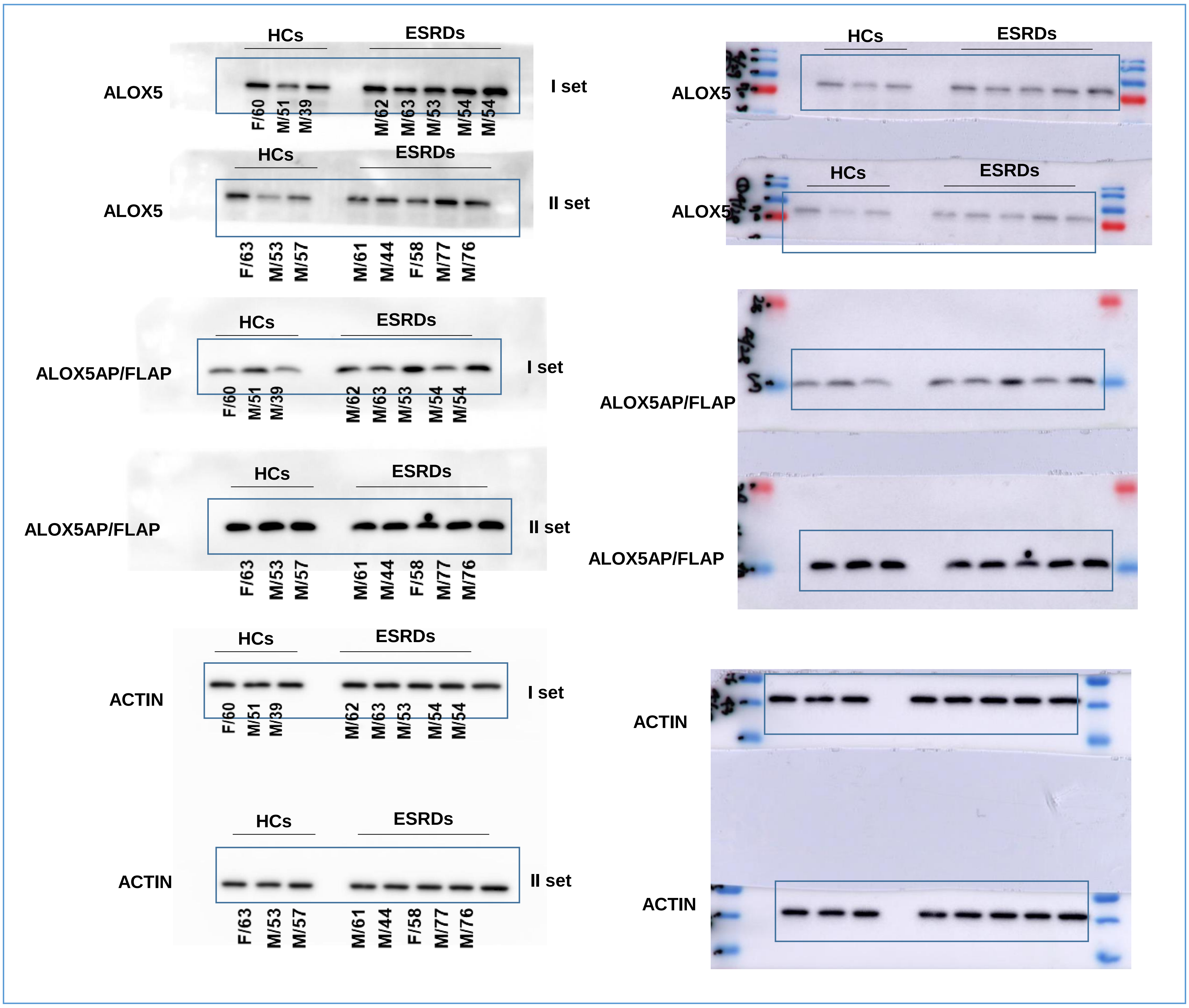

File name: Alox5_Alox5ap_mo.jpg
Actin_mo.jpg

Figure 6G, western blotting data

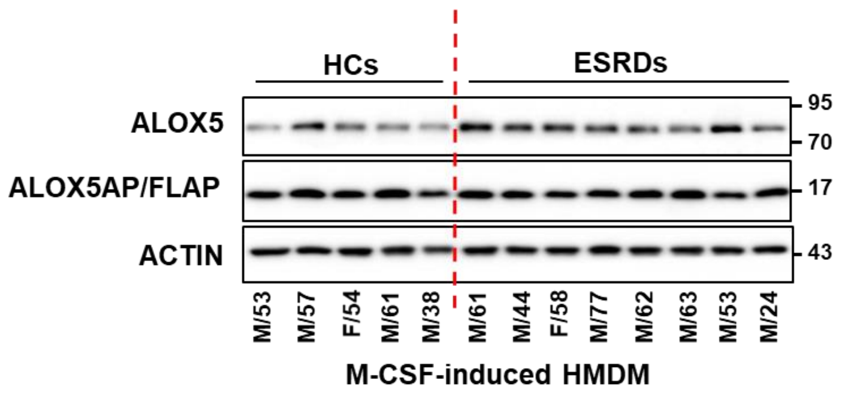

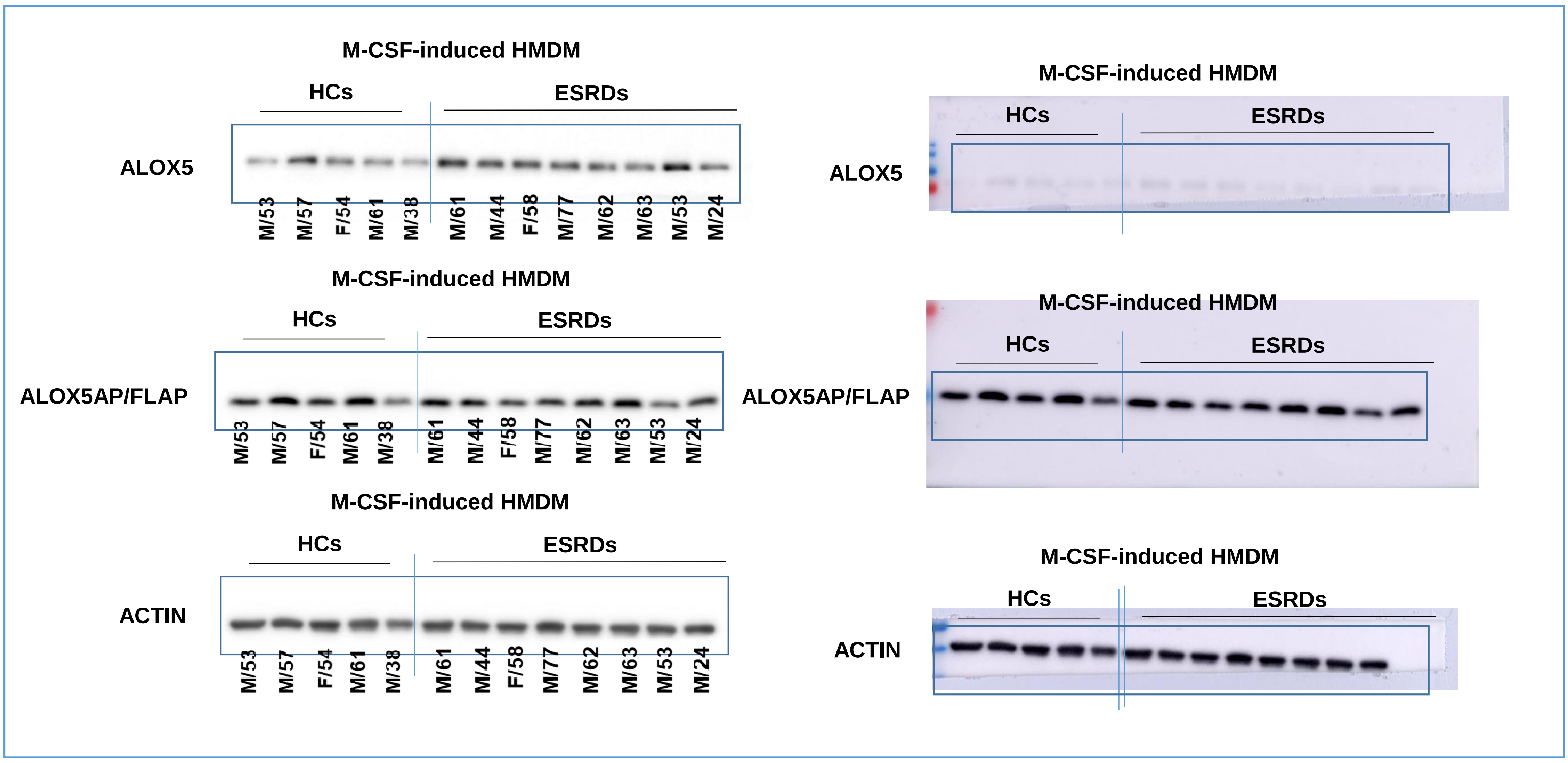

→ File name: ALOX5_HMDM.jpg
ALOX5AP_HMDM.jpg
Actin_HMDM.jpg

Figure 6L, western blotting data

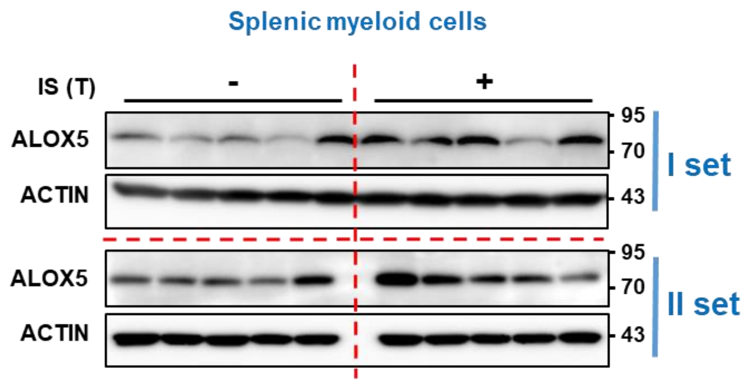

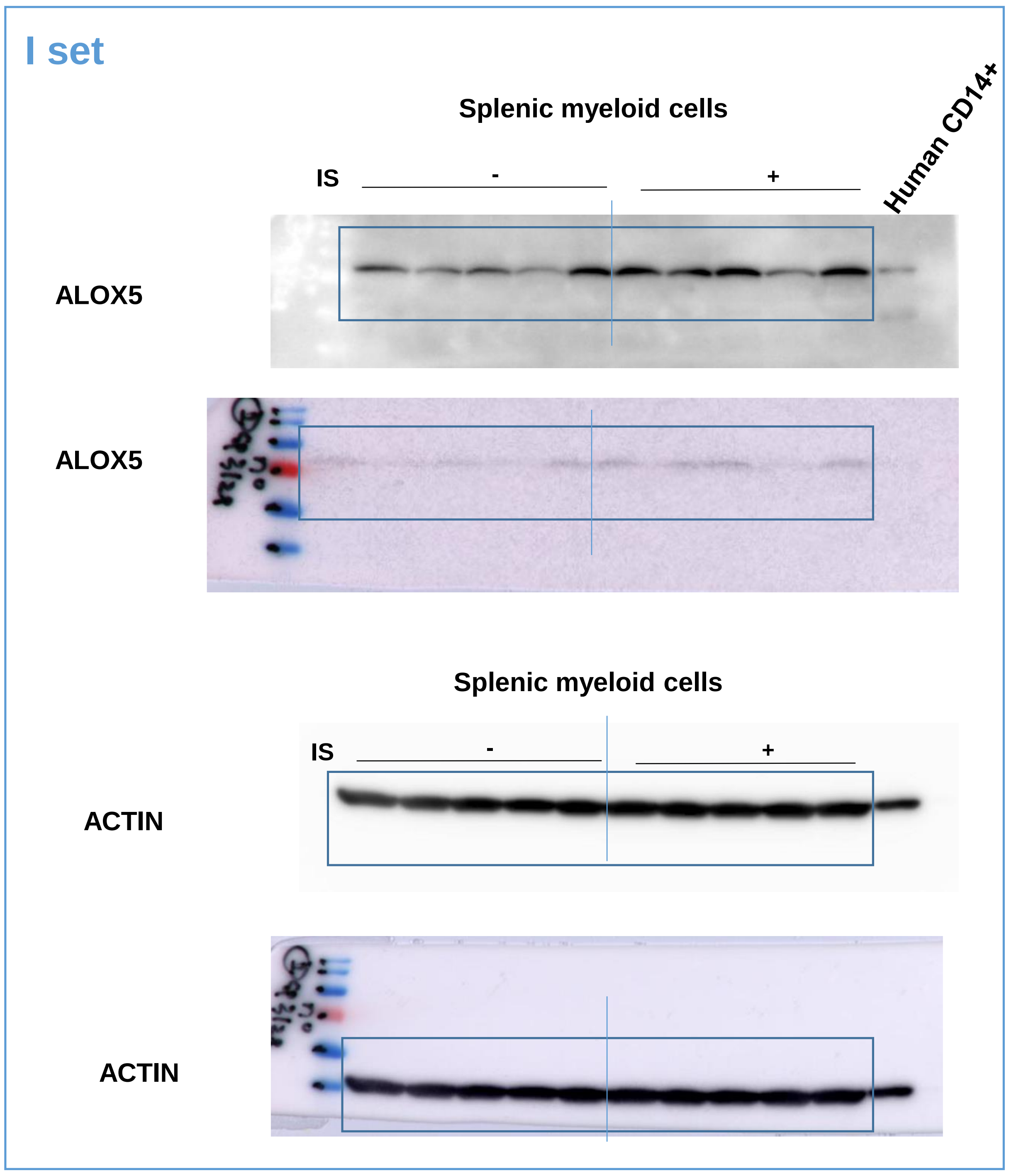

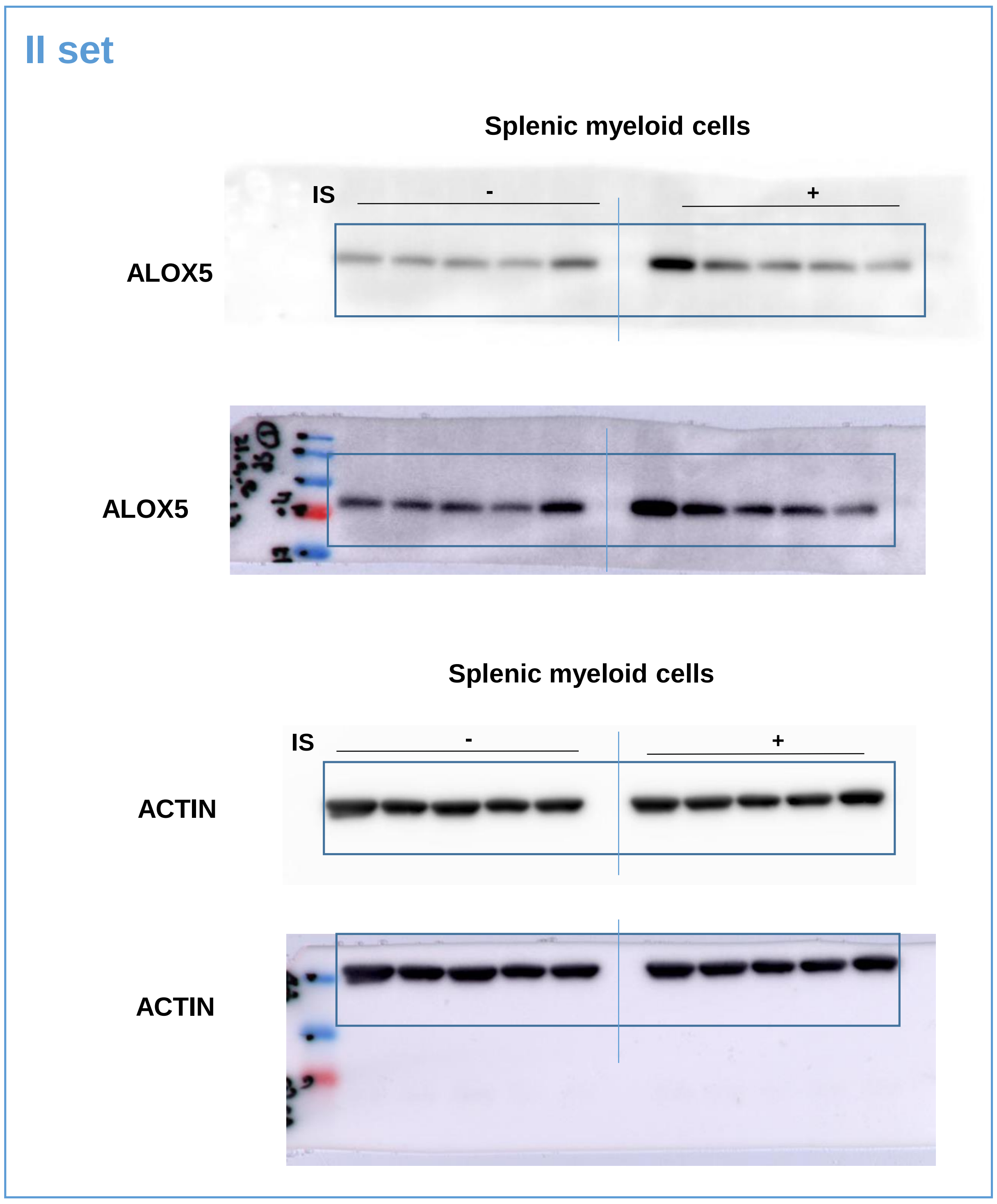

→ File name: mALOX5-1_sp.jpg
mActin-1_sp.jpg

→ File name: mALOX5_sp.jpg
mActin_sp.jpg
