# Supplementary material for: Uremic toxin indoxyl sulfate induces trained immunity via the AhR-dependent arachidonic acid pathway in end-stage renal disease (ESRD)
Source: eLife. 2024 Jul 9;12:RP87316. doi: 10.7554/eLife.87316 (PMC11233136; doi:10.7554/eLife.87316)
Supplement: Figure 6—figure supplement 1—source data 2. [file elife-87316-fig6-figsupp1-data2.pdf]

Figure 6-figure supplement 1E, western blotting data

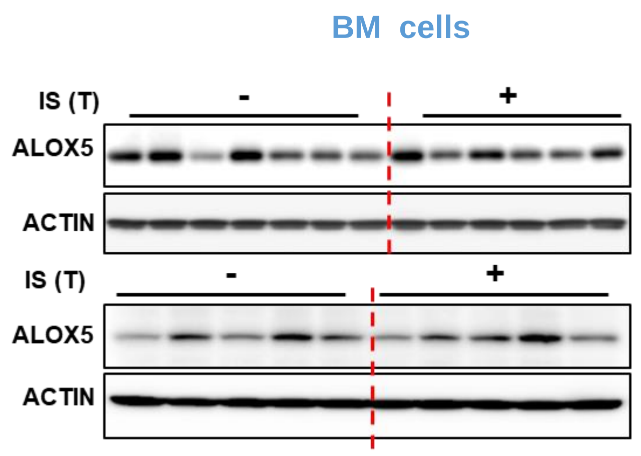

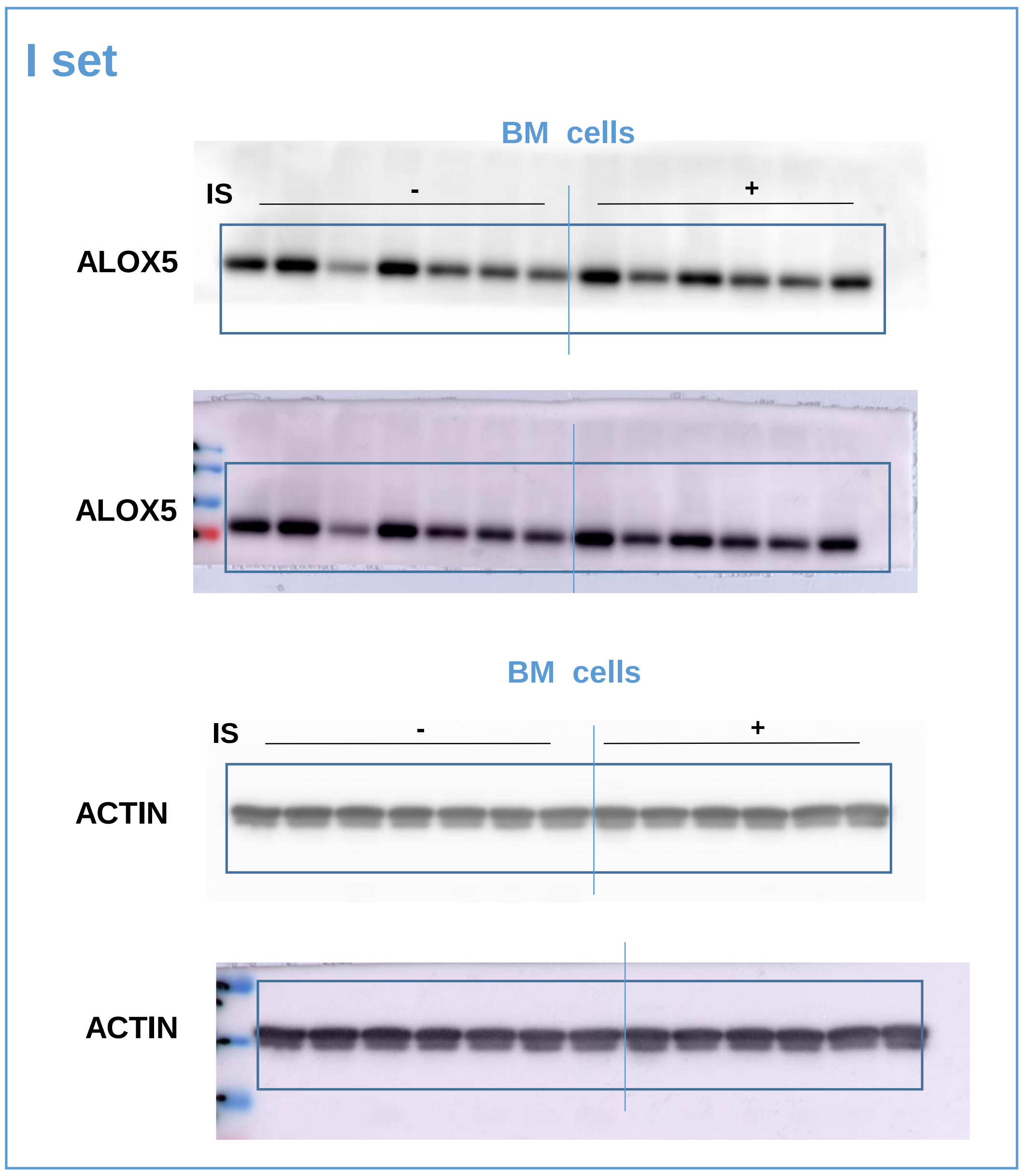

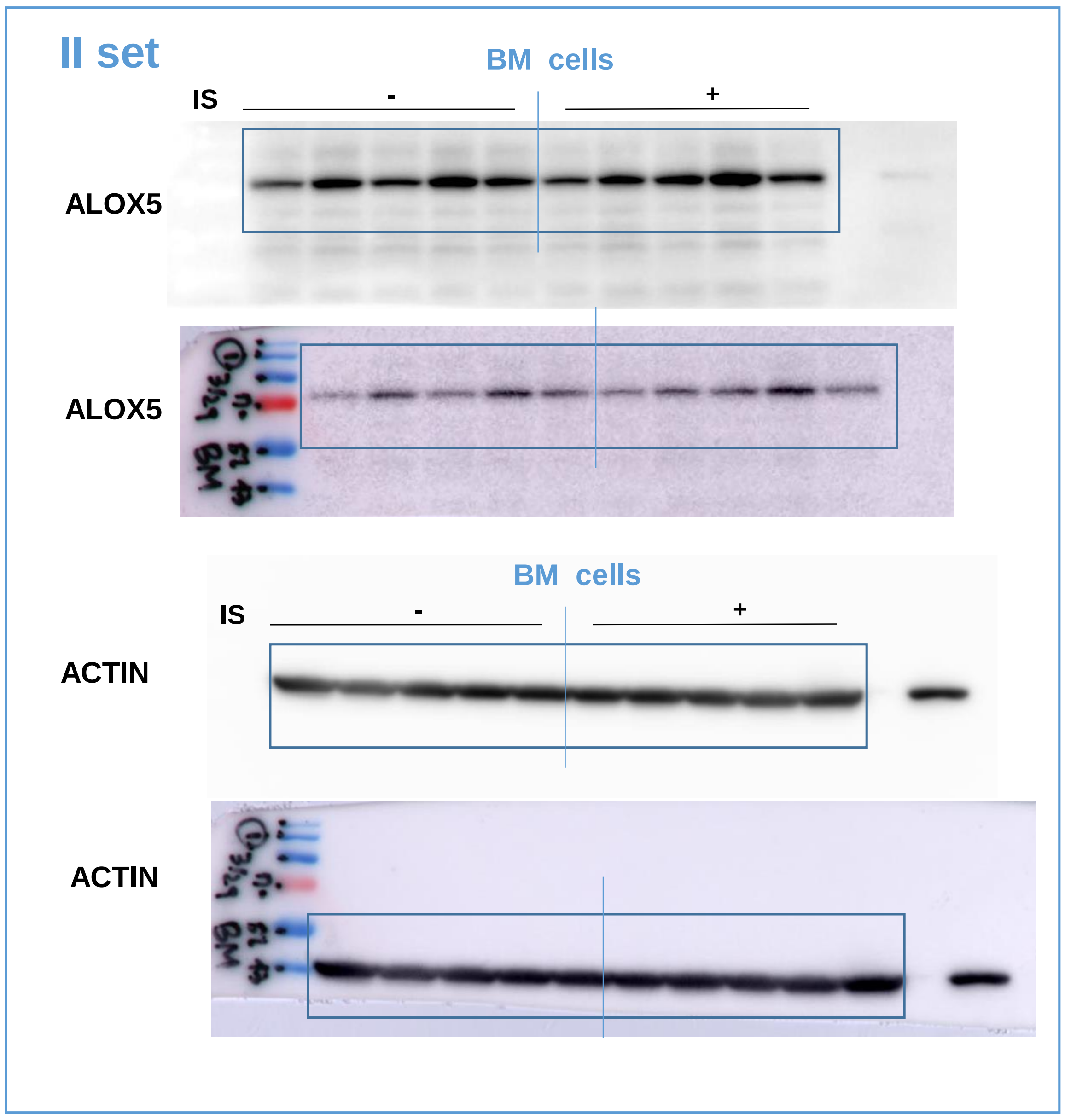

→ File name: Alox5_BM-1.jpg
Actin_BM-1.jpg

→ File name: Alox5_BM.jpg
Actin_BM.jpg
